# A novel partitivirus orchestrates conidiation, stress response, pathogenicity, and secondary metabolism of the entomopathogenic fungus *Metarhizium majus*

**DOI:** 10.1371/journal.ppat.1011397

**Published:** 2023-05-22

**Authors:** Ping Wang, Guogen Yang, Najie Shi, Cheng Zhao, Fenglin Hu, Robert H. A. Coutts, Ioly Kotta-Loizou, Bo Huang

**Affiliations:** 1 Anhui Provincial Key Laboratory of Microbial Pest Control, Anhui Agricultural University, Hefei, China; 2 Anhui Province Key Laboratory of Integrated Pest Management on Crops, School of Plant Protection, Anhui Agricultural University, Hefei, China; 3 Department of Life Sciences, Faculty of Natural Sciences, Imperial College London, South Kensington Campus, London, United Kingdom; Purdue University, UNITED STATES

## Abstract

Mycoviruses are widely present in all major groups of fungi but those in entomopathogenic *Metarhizium* spp. remain understudied. In this investigation, a novel double-stranded (ds) RNA virus is isolated from *Metarhizium majus* and named Metarhizium majus partitivirus 1 (MmPV1). The complete genome sequence of MmPV1 comprises two monocistronic dsRNA segments (dsRNA 1 and dsRNA 2), which encode an RNA-dependent RNA polymerase (RdRp) and a capsid protein (CP), respectively. MmPV1 is classified as a new member of the genus *Gammapartitivirus* in the family *Partitiviridae* based on phylogenetic analysis. As compared to an MmPV1-free strain, two isogenic MmPV1-infected single-spore isolates were compromised in terms of conidiation, and tolerance to heat shock and UV-B irradiation, while these phenotypes were accompanied by transcriptional suppression of multiple genes involved in conidiation, heat shock response and DNA damage repair. MmPV1 attenuated fungal virulence since infection resulted in reduced conidiation, hydrophobicity, adhesion, and cuticular penetration. Additionally, secondary metabolites were significantly altered by MmPV1 infection, including reduced production of triterpenoids, and metarhizins A and B, and increased production of nitrogen and phosphorus compounds. However, expression of individual MmPV1 proteins in *M*. *majus* had no impact on the host phenotype, suggesting insubstantive links between defective phenotypes and a single viral protein. These findings indicate that MmPV1 infection decreases *M*. *majus* fitness to its environment and its insect-pathogenic lifestyle and environment through the orchestration of the host conidiation, stress tolerance, pathogenicity, and secondary metabolism.

## Author summary

Fungi can carry mycoviruses, which are viruses that infect fungi. In this study, a new mycovirus called Metarhizium majus partitivirus 1 (MmPV1) was isolated from *Metarhizium majus*, a fungus that is well-known to kill insects. The virus has two segments of genetic material that code for a protein that helps replicate the virus (RNA-dependent RNA polymerase) and a protein that makes up the virus’s outer shell (capsid protein). Phylogenetic analysis showed that MmPV1 belongs to the *Gammapartitivirus* genus of the *Partitiviridae* family. This report revealed that MmPV1 infection decreased the fitness of the fungus in its environment by affecting its ability to produce spores, tolerate heat and UV-B radiation, and penetrate insect cuticles. This decreased pathogenicity was accompanied by changes in the production of secondary metabolites, including reduced production of certain compounds and increased production of others. Interestingly, expressing individual MmPV1 proteins did not have an impact on the host phenotype, indicating that the combination of viral proteins is responsible for the observed effects. Overall, this study highlights the complex interactions between mycoviruses and their fungal hosts and sheds light on the role of viruses in shaping fungal ecology and evoluti.

## Introduction

Mycoviruses or fungal viruses, first found in the cultivated mushroom *Agaricus bisporus* [1], are widely distributed mainly in filamentous fungi and yeasts [2]. Mycoviruses have different types of genomes including double-stranded (ds) RNA, positive-sense (+) and negative-sense (–) single-strand (ss) RNA, and ssDNA. Most reported mycoviruses have dsRNA genomes and are classified into 9 families and 1 unassigned genus: *Amalgaviridae*, *Chrysoviridae*, *Curvulaviridae*, *Megabirnaviridae*, *Partitiviridae*, *Polymycoviridae*, *Quadriviridae*, *Spinareoviridae*, *Totiviridae* and *Botybirnavirus* (https://ictv.global/taxonomy). Unlike viruses from hosts in other kingdoms, mycoviruses commonly lack an extracellular replication stage and are transmitted vertically (via asexual or sexual spores) or horizontally (*via* hyphal fusion or mating) [3]. However, some mycoviruses such as Sclerotinia sclerotiorum hypovirulence-associated DNA virus 1 (SsHADV-1) may be transmitted extracellularly [4] or via insect vectors [5], while others such as Magnaporthe oryzae chrysovirus 1-D (MoCV1-D) may be detected in cell-free culture supernatant [6].

The majority of mycoviruses are asymptomatic *in vitro*, but some studies demonstrate that mycovirus infection may lead to phenotypic alterations. For example, SsHADV-1 converted *S*. *sclerotiorum* from a plant pathogenic fungus to a beneficial endophyte [7]. Beauveria bassiana polymycovirus (BbPmV) 1 and 3 affected pigmentation, conidiation, and growth rate, as mediated by host metabolic pathways [8]. Similarly, several investigations showed that partitivirus infection specifically may cause phenotypic alterations. Fusarium equiseti partitivirus 1 (FePV1) decreased mycelial growth and biomass production [9], while mixed infection with of *Botryosphaeria dothidea* with Botryosphaeria dothidea chrysovirus 1 (BdCV1) and Botryosphaeria dothidea partitivirus 1 (BdPV1) was associated with attenuated mycelial growth, virulence and sectoring [10].

Mycoviruses also influence fungal secondary metabolisms. For instance, aflatoxin levels steadily increased by eradication of Penicillium chrysogenum virus (PcV) using dsRNA synthesis inhibitors [11], and aflatoxin production was inversely correlated to the presence of virus-like particles (VLPs) in *Aspergillus flavus* [12]. Conversely, a totivirus infecting *Magnaporthe oryzae* induced synthesis of the mycotoxin tenuazonic acid (TeA) [13] and Alternaria alternata chrysovirus 1 (AaCV1) increased AK-toxin levels in its host [14]. Additionally, resistance of *Penicillium digitatum* to the triazole drug prochloraz decreased in fungal strains co-infected with Penicillium digitatum polymycovirus 1 (PdPmV1) and Penicillium digitatum narna-like virus 1 (PdNLV1) [15]. MoCV1-D caused abnormal pigmentation and colony albinization through reduced accumulation of the melanin biosynthesis intermediate scylatone [6]. However, the effect of viral infection on the fungal metabolome of fungi has been rarely reported.

The entomopathogenic fungi *Metarhizium* spp., infect hundreds of insect species worldwide and play a key role in agricultural and forest pest control. *Metarhizium* strains do not only act as biocontrol agents against pests but also increase plant fresh weight [16]. Mycoviruses in *Metarhizium* spp. include the first reported *Metarhizium* dsRNAs, evidently encapsidated in VLPs in 2 of 41 *M*. *anisopliae* isolates examined [17]. Following sub-culturing, both the dsRNAs and VLPs disappeared, but no difference in virulence was observed between virus-infected and virus-free strains. Further studies revealed that mycoviral infection may not be latent in *Metarhizium*, since mycoviruses isolated from *M*. *anisopliae* and *M*. *anisopliae* var. *acridum* decreased mycelial growth, conidial production and virulence [18,19]. Additionally, mycoviral infection in *M*. *anisopliae* enhanced endochitinase secretion [20]. Many *Metarhizium* spp. mycoviruses have been reported and characterized by the Research Center for Entomogenous Fungi of Anhui Agricultural University (RCEF). These include Metarhizium brunneum partitivirus 1 (MbPV1) and 2 (MbPV2), classified respectively to the genera *Epsilonpartitivirus* and *Gammapartitivirus* in the family *Partitiviridae* [21,22], and the unassigned Metarhizium brunneum bipartite mycovirus 1 (MbBV1) [23]. There is evidence that *Metarhizium* spp. contain large numbers of mycoviruses, which may not be completely latent [18]. Nevertheless, in-depth knowledge of *Metarhizium* mycoviruses and their effects on host phenotype remains limited.

Here, we describe and characterize a novel mycovirus Metarhizium majus partitivirus 1 (MmPV1). MmPV1 was found to have pleiotropic effects on the host lifecycle. Our study expands knowledge of the mycovirus diversity and function in *M*. *majus*, and provides novel insights into virus-fungus interactions.

## Results

### Partitivirus MmPV1 comprises two dsRNA segments

**Fig 1:**
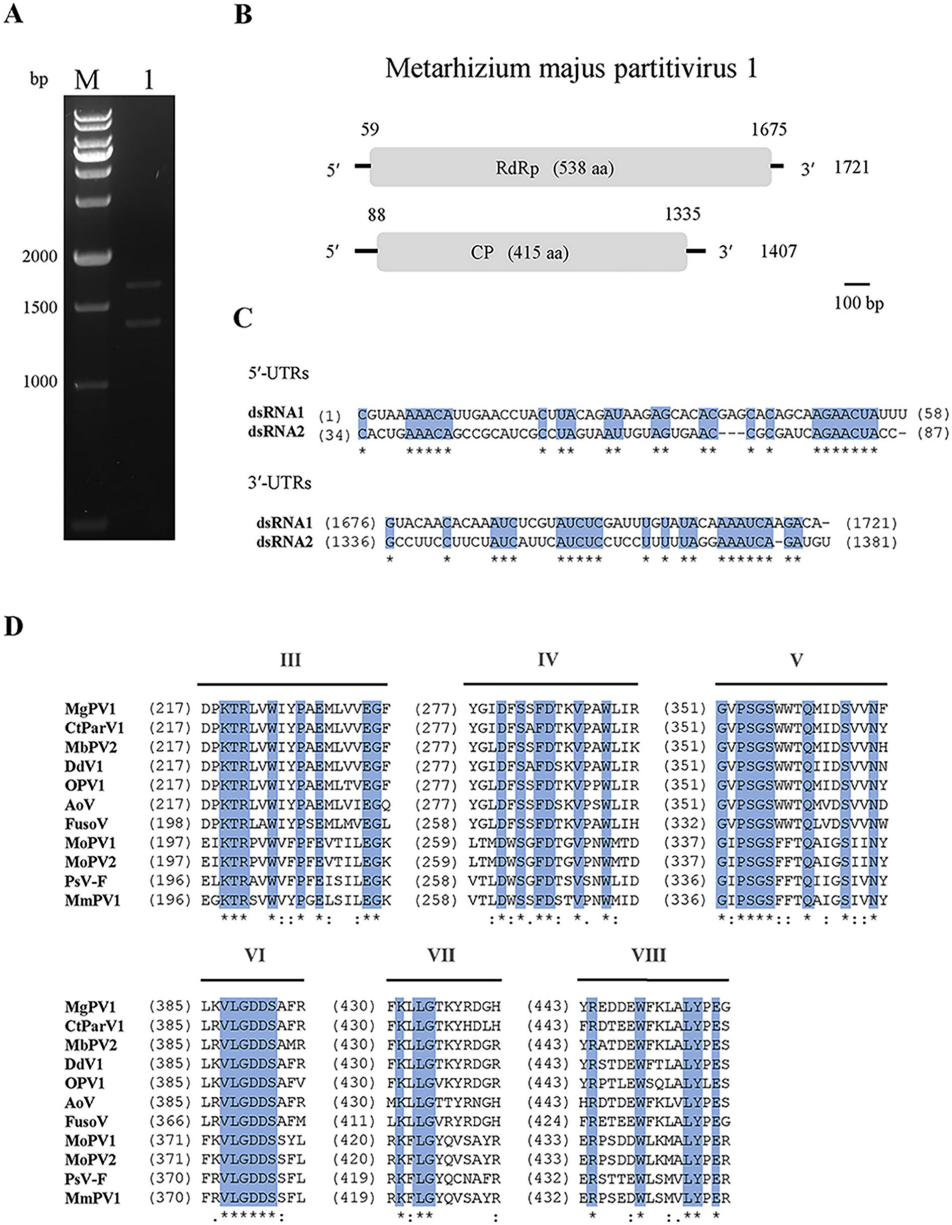
Characterization of Metarhizium majus partitivirus 1 (MmPV1). (A) Electrophoretic profiles of MmPV1 in RCEF0578. M, DNA molecular weight marker; lane 1, dsRNAs of MmPV1. (B) Schematic representation of the genomic organisation of MmPV1. (C) The 5′- and 3′-terminal nucleic acid sequences of MmPV1. The shaded areas represent 100% nucleotide identity. (D) Multiple amino acid sequence alignment of the putative RdRp of MmPV1 and ten other gammapartitiviruses. The shaded areas indicate a position with a single, fully conserved residue.

Two dsRNA segments *ca*. 1.7 and 1.4 kbp in size were discovered in *M*. *majus* strain RCEF0578 (Fig 1A), originally isolated from a stick insect in Anhui Province, China.

The full-length sequences of MmPV1 dsRNAs 1 and 2 were determined by combining RNA-sequencing and RLM-RACE. The complete genome of MmPV1 consists of dsRNA1, 1721 bp in length (accession number OL518956), and dsRNA2, 1407 bp in length (accession number OL518957), with G+C contents of 45% and 47.4%, respectively. Each dsRNA contains a single ORF, encoding putative proteins 538 and 415 aa in length, or 62.55 kDa and 46.31 kDa in size, respectively (Fig 1B). The 5′-terminal sequences of dsRNA1 and dsRNA2 are 58 and 87 bp in length and the corresponding 3′-terminal sequences are 46 and 72 bp in length, respectively. Sequence analysis showed that 5′-termini possess a conserved region AGAACUA, while 3′-termini contain another conserved region UNUNUANNAAAUCANGA (Fig 1C).

The putative proteins encoded by dsRNAs 1 and 2 shared sequence similarities of 69.54% and 59.23% with respectively the RdRp (ATD50490.1) and the CP (ATD50491.1) of Magnaporthe oryzae partitivirus 2. Conserved domain analysis revealed that the protein encoded by dsRNA1 features a RdRp domain (RdRp_1; pfam00680) including a C-terminal domain (90–451 aa) and a catalytic domain (255–389 aa). The RdRP domain possesses six conserved motifs (III-VII) common within the genus *Gammapartitivirus* (Fig 1D).

**Fig 2:**
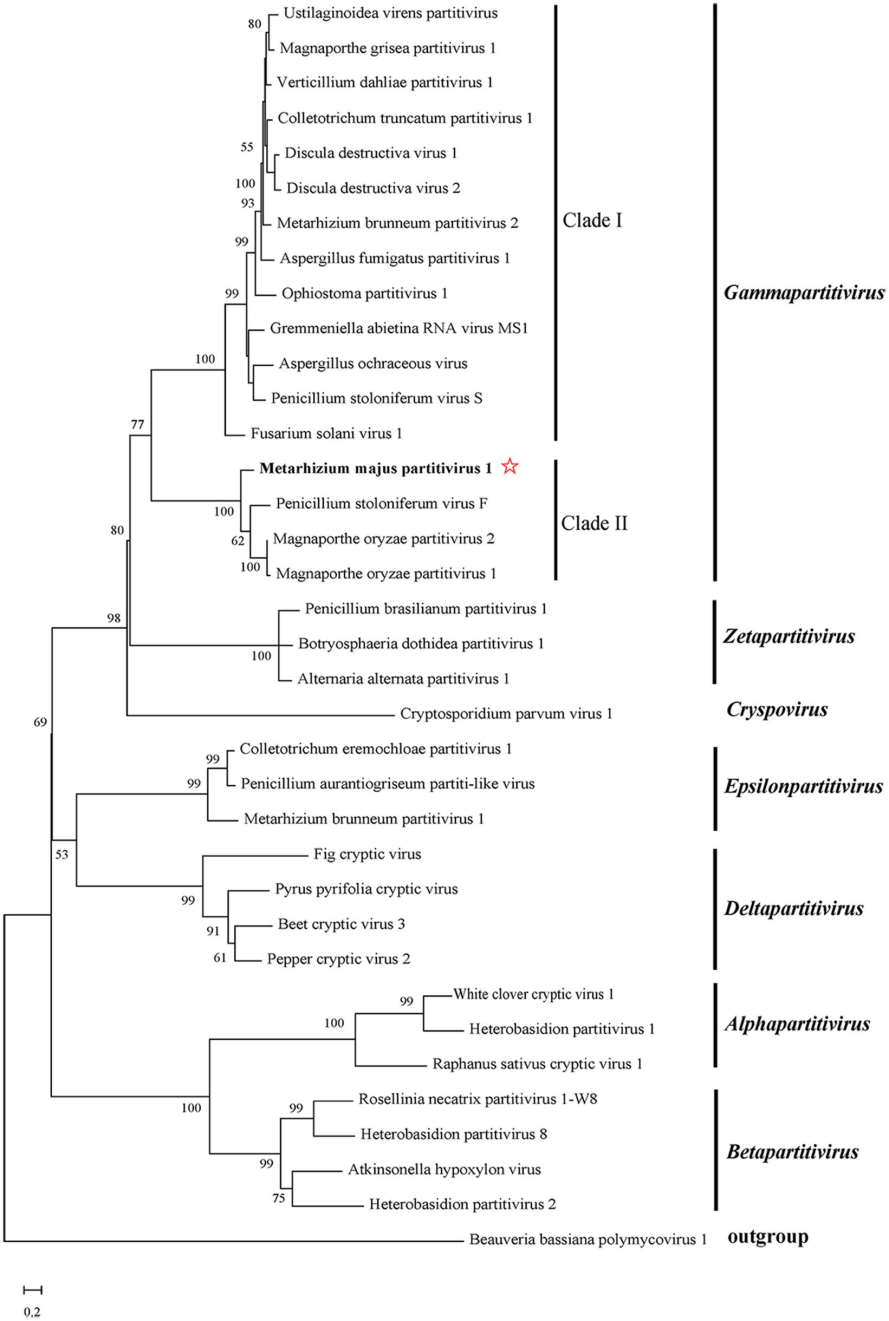
Phylogenetic analysis of MmPV1. The ML phylogenetic tree based on RdRp sequences of 34 partitiviruses including MmPV1 and representatives from seven genera of the family *Partitiviridae*, (Table S1), with Beauveria bassiana polymycovirus 1 (BbPmV-1) as the outgroup.

Phylogenetic analysis using *Partitiviridae* RdRp sequences and including Beauveria bassiana polymycovirus 1 (BbPmV-1; YP_009352879.1) as an outgroup revealed that MmPV1 clustered with 16 gammapartitiviruses and was distinguishable from the other six genera of the family (Fig 2). The threshold criteria for belonging to the genus gammapartitivirus are 90% and 80% similarity for respectively the RdRp and the CP [24]. The phylogenetic analysis confirmed that MmPV1 is a new member of the genus *Gammapartitivirus* in the family *Partitiviridae*.

### MmPV1 is transmitted horizontally

**Fig 3:**
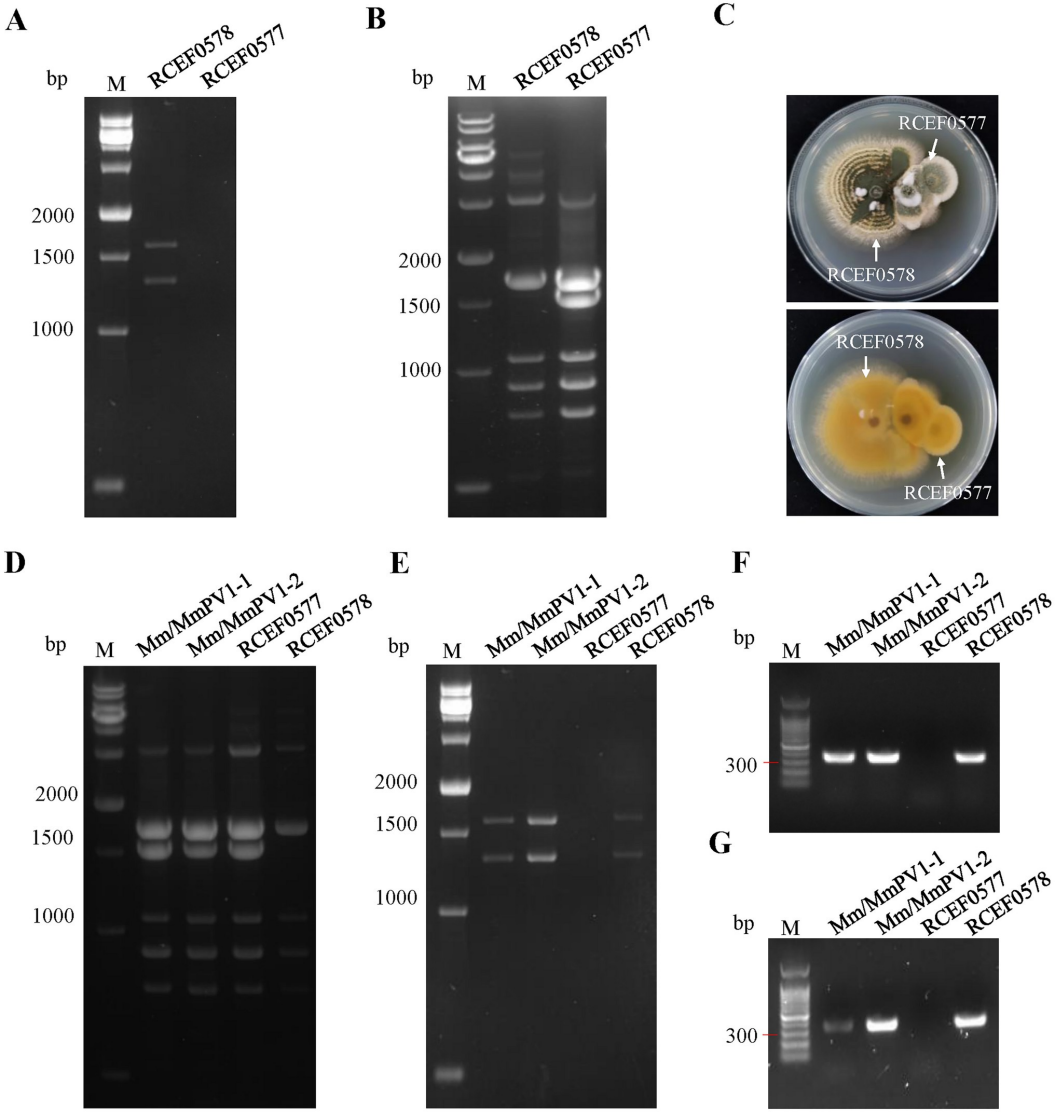
Horizontal transmission of MmPV1. (A) MmPV1 dsRNA segments in RCEF0578 and RCEF0577. (B) ISSR-PCR with primer P9 of *M*. *majus* isolates. (C) Dual-culture of *M*. *majus* isolates, the front of the colony (above) and the back of the colony (below). (D) Confirmation of MmPV1 horizontal transmission in RCEF0577 strains with ISSR primer P9. (E) dsRNAs extraction from RCEF0577 strains. (F) RT-PCR of dsRNA with MmPV1 specific primers. (G) RT-PCR of total RNA with specific primers ORF1-F/ORF1-R of MmPV1.

The *M*. *majus* virus-free strain RCEF0577 (Fig 3A) was identified by ISSR-PCR with ten primers (M1, M10, M15, M17, P8, P9, P11, P12, 889 and 850) (S1C, S1D and S1E Fig) and used as a recipient for horizontal transmission of MmPV1. ISSR-primers P9, 889 and 850 were confirmed as appropriate to distinguish RCEF0577 from RCEF0578, since the amplification products, approximately 1,500, 1,750, and 2,000 bp in size, are different in the two strains (Fig 3B and S1D and S1E Fig).

Although a demarcation line along the zone of contact was observed during co-cultivation of RCEF0577 and RCEF0578 indicating vegetative incompatibility (Fig 3C), two isogenic strains were obtained through within-species transmission. A total of 56 single-spore isolates of *M*. *majus* were obtained, and 24 of them were identified as RCEF0577 by ISSR-PCR (S2A Fig). Subsequently, dsRNA isolation and RT-PCR amplification were employed to confirm the presence of MmPV1 (Fig 3D–3G); two isogenic strains containing MmPV1, namely Mm/MmPV1-1 and Mm/MmPV1-2, were selected for further assays (S2B Fig). These results indicated that MmPV1 could be horizontally transmitted into another M. majus strain which is potentially vegetatively incompatible to its original host.

### MmPV1 significantly decreases *M*. *majus* conidiation

**Fig 4:**
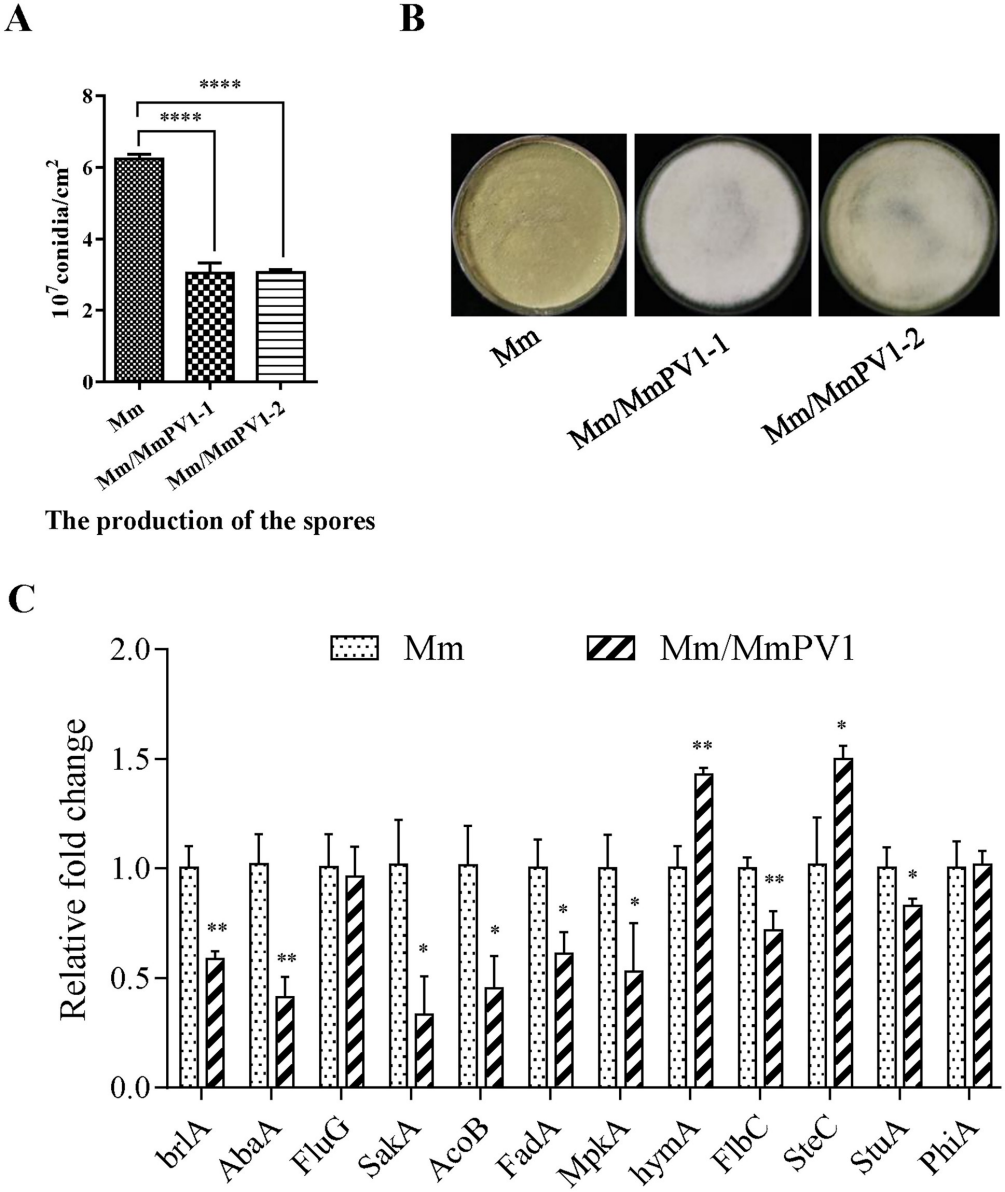
Effect of MmPV1 on conidiation and growth of *M*. *majus*. (A) Conidiation of Mm and Mm/MmPV1 strains cultured on PDA for 14 d, together with colony morphology. (B) Colony morphology of different strains cultured on PDA plates for 14days. (C) Relative expression levels of conidiation-related genes as shown by qRT-PCR. ANOVA *, *P* <0.05; **, *P* <0.01; ****, *P* <0.0001.

There were no significant differences in the radial growth of Mm/MmPV1-1 and -2 as compared to Mm on PDA, SDAY and 1/4 strength SDAY (S3 Fig). However, following growth on PDA the conidial yields for Mm, Mm/MmPV1-1 and Mm/MmPV1-2 were 6.24 ± 0.08, 3.04 ± 0.16 and 3.05 ± 0.05 (10^7^ conidia cm^-2^), respectively (Fig 4A and 4B). The conidiation capacity of both Mm/MmPV1-1 and -2 was significantly decreased by more than 50% as compared to Mm. Moreover, 8 out of 12 genes involved in conidiation of filamentous fungi were downregulated in Mm/MmPV1 strain, including the asexual developmental activator genes *brlA* and *abaA*, the mitogen-activated kinase (MAPK) gene *sakA*, and the genes encoding COP9 signalosome subunit 7 *acoB*, G protein alpha subunit *fadA*, protein kinase-like domain protein *mpkA*, C2H2 finger domain protein FlbC *flbC* and APSES transcription factor *stuA* (Fig 4C). These results indicated a marked effect of MmPV1 on conidiation but no detectable impact on the vegetative growth of *M*. *majus*.

### MmPV1 reduces tolerance to heat shock and UV-B irradiation

**Fig 5:**
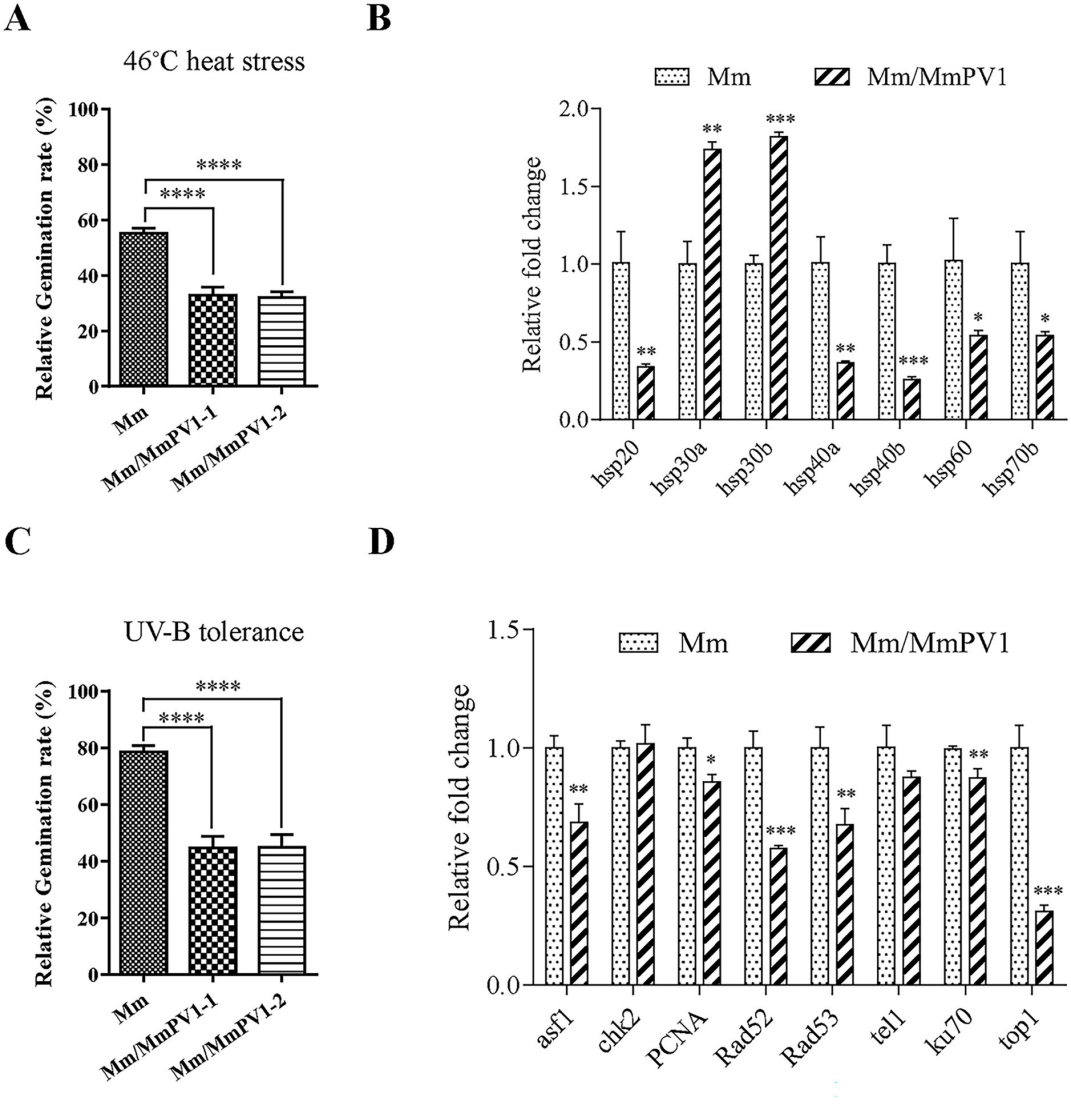
Effect of MmPV1 on heat shock and UV-B irradiation tolerance. (A) Relative germination rates of Mm and Mm/MmPV1 strains following heat shock treatment for 24 h. (B) Relative expression levels of genes involved in response to heat shock as shown by RT-qPCR. (C) Relative germination rates of different strains following UV-B irradiation treatment for 24 h. (D) Relative expression levels of genes involved in DNA damage repair genes as shown by qRT-PCR. ANOVA *, *P* <0.05; **, *P* <0.01; ***, *P* <0.001; ****, *P* <0.0001.

Despite GT_50_ values not being affected under normal culture conditions (S4 Fig), the germination rates of Mm/MmPV1 under heat stress were significantly lower as compared to Mm (Fig 5A and S2 Table). Concomitant with these reactions to heat shock, 5 out of 7 heat shock-responsive genes HSP20-like chaperone (*hsp20*), heat shock protein DnaJ (*hsp40a*), DnaJ domain containing protein (*hsp40b*), heat shock protein 60 (*hsp60*) and heat shock protein 70 (*hsp70b*) were downregulated by 45–75% in the presence of MmPV1 (Fig 5B). Conversely, the expression of heat shock protein 30 (*hsp30a*, *hsp30b*) was upregulated almost 2-fold in Mm/MmPV1 (Fig 5B).

After UV-B irradiation, the relative germination rate was decreased *ca*. 43% for Mm/MmPV1 as compared to Mm, (Fig 5C and S2 Table), and this reduction was accompanied by suppressed expression of several genes involved in DNA damage repair. (Fig 5D).

Finally, no difference between Mm/MmPV1 and Mm was noted in their responses to osmotic, oxidative and cell wall perturbing stresses (S5 Fig).

### MmPV1 attenuates virulence during cuticle infection

**Fig 6:**
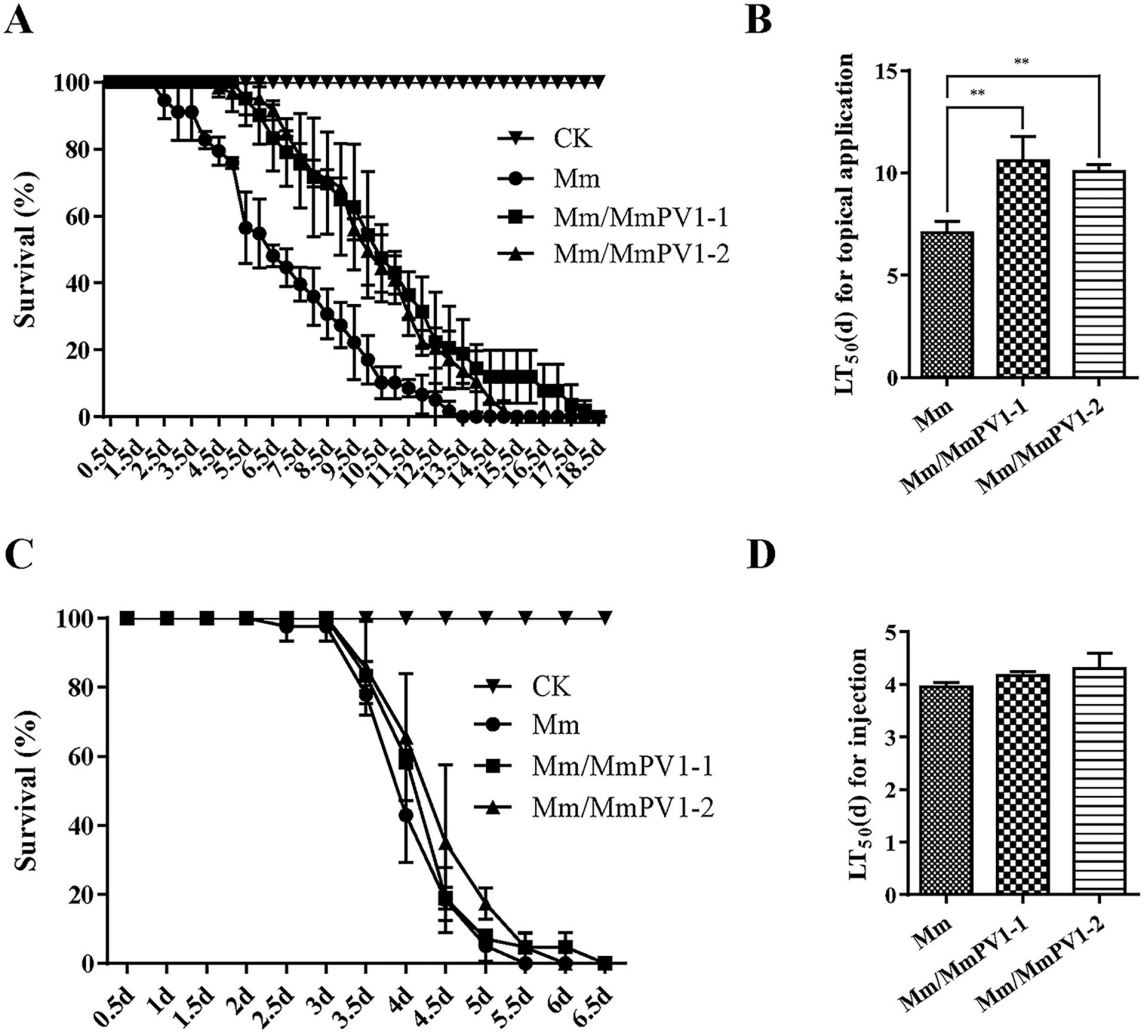
Effect of MmPV1 on fungal virulence. (A) Survival rates of *G*. *mellonella* larvae following injection with conidial suspensions from Mm and Mm/MmPV1 strains. Control insects were treated with sterile water. (B) Mean lethal times (LT_50_) of different strains after injection. (C) Survival rates of *G*. *mellonella* larvae following topical infection with conidial suspensions from different strains. Control insects were treated with sterile water. (D) The mean lethal times (LT_50_) of different strains after topical application. **, *P* <0.01.

The survival rates of *G*. *mellonella* larvae following topical infection with Mm/MmPV1 were higher than with Mm (Fig 6A), resulting in LT_50_ estimates of 10.59 ± 0.70 and 10.07 ± 0.20 days for Mm/MmPV1-1 and -2 as compared to 7.04 ± 0.33 days for Mm (Fig 6B). However, the LT_50_ estimates *via* cuticle-bypassing infection (injection) revealed no significant differences between Mm and Mm/MmPV1 (Fig 6C–6D). These results indicate that MmPV1 attenuates virulence during the cuticle infection phase.

### MmPV1 decreases conidial hydrophobicity, adhesion and penetration ability

**Fig 7:**
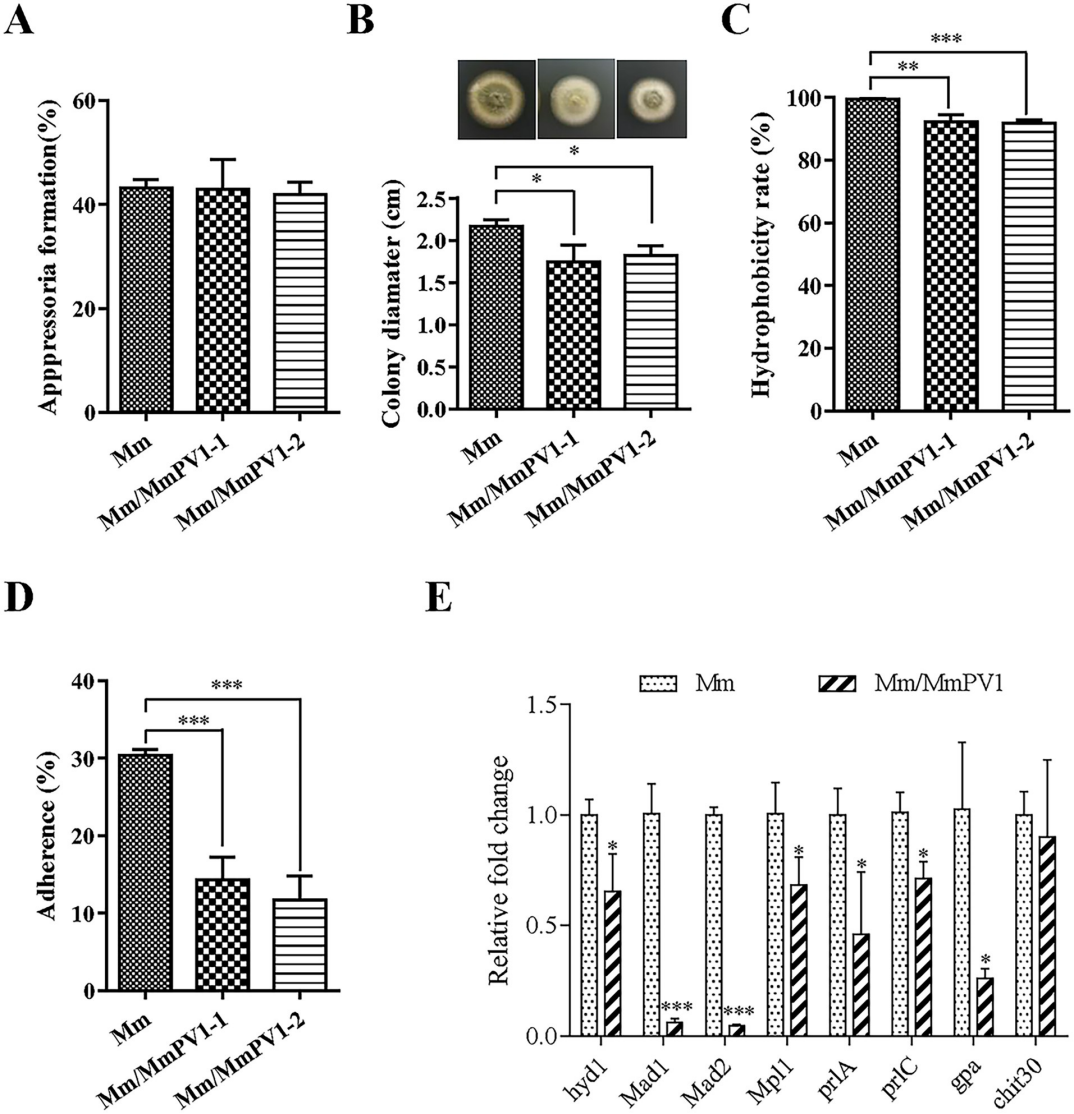
Analysis of fungal virulence-related phenotypes. (A) Appressorium formation rate of Mm and Mm/MmPV1 strains in plastic hydrophobic plate. (B) Colony diameter of different strains following penetration of the wings of *C*. *atrata* and subsequent culturing for 4 d, together with colony morphology. (C) Hydrophobicity of different strains. (D) Adherence of different strains in hydrophobic plate. (E) Relative expression levels of virulence-related genes as shown by qRT-PCR. ANOVA *, *P* <0.05; **, *P* <0.01; ***, *P* <0.001.

Conidial hydrophobicity was significantly reduced in Mm/MmPV1 as compared to Mm, with hydrophobicity measurements of 99.54% ± 0.08%, 92.25% ± 1.25% and 91.75% ± 0.63% for respectively Mm, Mm/MmPV1-1 and Mm/MmPV1-2, indicating that MmPV1 reduces host hydrophobicity (Fig 7C). Conidial adhesion was also significantly lower in Mm/MmPV1 as compared to Mm (Fig 7D). There were no differences in appressorium formation rates between Mm and Mm/MmPV1 (Fig 7A), suggesting no impact of MmPV1 on the appressorium, which is involved in cuticle penetration [25]. However, Mm formed significantly larger colonies on cicada wings than Mm/MmPV1-1 and -2 (Fig 7B), suggesting an impact of the viral infection on the ability of the fungus to penetrate insect cuticles.

The expression levels of several virulence-related genes involved in cuticle penetration (*pr1A*, subtilisin-like protease Pr1A; *pr1C*, subtilisin-like serine protease PRlC), hydrophobicity (*hyd1*, hydrophobin) and adhesion (*mad1*, adhesin protein Mad1; *mad2*, adhesin protein Mad2) were all downregulated in Mm/MmPV1 (Fig 7E). Therefore, we speculate that MmPV1 attenuates the virulence of *M*. *majus* by decreasing conidial hydrophobicity, adhesion and cuticular penetration.

### MmPV1 alters production of metabolites

**Fig 8:**
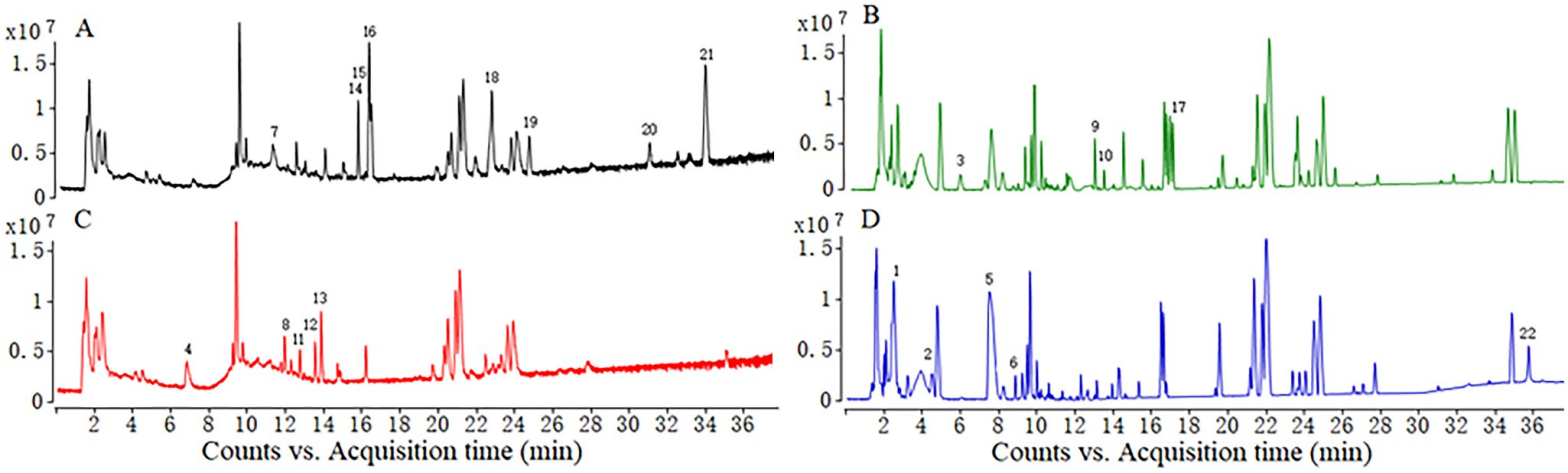
Total ion flow chromatograms of extracts from MmPV1-infected and -free *M*. *majus*. (A) The MmPV1-free strain in negative model. (B) The MmPV1-free strain in positive model. (C) The MmPV1-infected strain in negative model. (D) The MmPV1-infected strain in positive model. Number 1 to 22 are the main metabolites which changed more than two times. There are marked on the relative bigger peaks.

HPLC-HRMS analysis revealed that 28 and 25 major metabolites were present respectively in Mm and Mm/MmPV1 (Fig 8 and S6 Fig). The levels of 22 metabolites were more than doubled (Table 1). For instance, the production levels of 10 metabolites in Mm/MmPV1 were increased as compared to Mm. Also, 6 major metabolites present in Mm/MmPV1 were not detected in Mm, suggesting that MmPV1 might be involved in fungal secondary metabolism.

**Table 1:** The metabolites changed more than two times in virus-free and virus-infected *Metarhizium majus* strains.

No.	Rt (Min)	Accurate mass	Pick area (x10^5^ counts. s)		lmax (nm)	Molecular formula	Possible compound
			Virus-free strain	Virus-infected strain			
1	2.48	656.3156	0	**1335.10±9.51**		C_25_H_49_N_6_O_12_P	unknown
2	4.50	640.3207	0	**317.38±3.08**		C_25_H_49_N_6_O_11_P	unknown
3	5.74	280.1019	**194.73±1.75**	0		C_8_H_16_N_4_O_7_	unknown
4	6.82	560.2833	0	**33.45±0.61**		C_27_H_44_O_12_	unknown
5	7.43	280.1423	789.49±5.39	**2322.78±13.05**	195, 225, 275	C_14_H_20_N_2_O_4_	*N*-(2-Amino-3-methylbutanoyl tyrosine
6	8.85	464.2370	40.67±0.76	**123.31±1.62**		C_20_H_36_N_2_O_10_	unknown
7	11.13	180.0423	**499.16±3.58**	93.39±1.20	225, 252, 338	C_9_H_8_O4	cordytropolone
8	11.91	478.1475	19.40±0.35	**107.22±1.31**	228, 276	C_23_H_26_O_11_	5-Acetyl-3,4-dihydro-6,8-dihydroxy-3-(5-oxo-1,3-pentadienyl)-1H-2-benzopyranone-6-methoxy-8-O-β-D-glucopyranoside
9	12.41	564.0751	**257.55±2.95**	0		C_24_H_20_O_16_	unknown
10	12.82	564.0751	**95.90±1.32**	0		C_24_H_20_O_16_	unknown
11	12.91	434.1213	0	**67.07±0.84**	226, 278, 400	C_21_H_22_O_10_	norlichexanthone-6-O-b -(4-O-methylglucopyranoside)
12	13.84	450.1526	0	**113.06±1.28**	230, 280, 330, 400	C_22_H_26_O_10_	indigotide B
13	14.25	450.1526	17.78±0.29	**197.94±2.05**	230, 280, 330, 400	C_22_H_26_O_10_	indigotide C
14	15.55	436.1733	**128.34±1.36**	0	230, 290	C_22_H_28_O_9_	aspertetranone D
15	15.65	422.1577	**128.32±1.31**	0		C_21_H_26_O_9_	indigotide A
16	16.19	422.1577	**507.63±3.78**	105.51±1.27	230, 340	C_21_H_26_O_9_	Isomer of indigotide A
17	16.84	442.2719	**369.55±3.10**	0		C_27_H_38_O_5_	Subglutinol C
18	22.63	428.2927	**536.80±3.67**	14.72±0.28		C_27_H_40_O_4_	metarhizins A
19	24.57	426.2770	**220.00±2.19**	0		C_27_H_38_O_4_	metarhizins B
20	30.85	470.3032	**141.74±1.85**	0		C_29_H_42_O_5_	Camphoratin G
21	33.7	484.3189	**819.82±6.03**	0		C_30_H_44_O_5_	Camphoratin F
22	35.7	781.5677	0	**268.97±2.24**		C_40_H_75_N_7_O_8_	unKnown

Two triterpenoids (compounds 20 and 21), with structures similar to the *Ganoderma lucidum* triterpenoids, were absent from Mm/MmPV1 [26], while the synthesis of metarhizins A and B (compounds 17 and 18) [27] decreased significantly in Mm/MmPV1 as compared to Mm. A total of 8 potentially unknown compounds were detected, while compound 4 and compound 3 were respectively present in Mm/MmPV1 and Mm. Interestingly, 5 out of 8 unknown compounds are nitrogen and phosphorus based, including compounds 1, 2, and 22 that were only detected in Mm/MmPV1; the production of compound 6 in Mm/MmPV1 was 3-fold higher than in Mm. These observations revealed that MmPV1 orchestrates metabolite synthesis.

### MmPV1 ORF1 or ORF2 do not alter host phenotypes

The *M*. *majus* strains expressing the MmPV1 ORFs, namely Mm-ORF1 and Mm-ORF2, were generated and confirmed using RT-PCR, RT-qPCR and immunoblotting (S7 Fig). The conidial yields for the Mm, Mm-ORF1, and Mm-ORF2 strains were found to be similar with counts of 6.10 ± 0.07, 5.76 ± 0.11, and 6.10 ± 0.14 (1×10^7^ conidia cm^-2^), respectively, as compared to Mm/MmPV1 with 2.41 ± 0.18 (1×10^7^ conidia cm^-2^) (S8 Fig). Similarly, the relative germination rates of Mm-ORF1 and Mm-ORF2 were not significantly different from Mm when tested for tolerance to heat shock and UV-B irradiation (S9 Fig). Furthermore, there were no significant differences in the LT_50_ values between the Mm isolate and Mm-ORF1 and Mm-ORF2 strains following either topical application or injection of *G*. *mellonella* larvae (S10 Fig). These results indicated that the presence and expression of a single MmPV1 ORF, either ORF1 or ORF2, did not by itself induce any MmPV1-mediated phenotypes.

## Discussion

*Partitiviridae* is a family of bisegmented dsRNA viruses that comprises five established genera *Alphapartitivirus*, *Betapartitivirus*, *Gammapartitivirus*, *Deltapartitivirus* and *Cryspovirus* [28] and two additional proposed genera “*Epsilonpartitivirus*” and “*Zetapartitivirus*”) [24,29]. The mycovirus found in M. majus RCEF0578 was identified as a new member of genus *Gammapartitivirus*. Phylogenetic analysis showed that members of genus *Gammapartitivirus* clustered in two clades, Clade I and Clade II, whose RdRp C-terminal domains consist of 420–450 aa in Clade I, or 320–380 aa in Clade II. MmPV1 was closely related to Penicillium stoloniferum virus F, and Magnaporthe oryzae partitivirus 1 and 2 in Clade II (Fig 2), and similar to its RdRp homologs in genus *Gammapartitivirus* contained six conserved motifs (Fig 1D).

Most mycoviruses lack an extracellular stage in their life cycles, with some exceptions such as Sclerotinia sclerotiorum hypovirulence associated DNA virus 1 [30], and spread through vertical (sporogenesis) and horizontal (cell fusion) transmission in nature [31]. A prerequisite for understanding fungal traits associated with mycoviral infection is the generation and comparison of isogenic lines with and without virus infection. Presently, three major methods are used to acquire isogenic strains, vertical transmission, horizontal transmission, and VLP transfection. Vertical transmission of mycoviruses mainly depends on a single-spore isolation method [32,33], sometimes combined with treatment with the protein synthesis inhibitor cycloheximide [34]. In the present study, this method was adopted to obtain isogenic strains from the host of MmPV1, and subsequent dsRNA extraction from hundreds of isolates confirmed that MmPV1 was transmitted to all conidial progeny. Single-spore isolation failed to obtain isogenic strains in previous cases of partitivirus infection, suggesting persistence [35]. Thus, we attempted horizontal transmission to obtain isogenic strains by means of co-cultivation. Due to a difficulty in distinguishing between the colony morphology of donor and recipient strains, resistance genes (for example, hygromycin and geneticin-resistance gene) may be utilized to screen the strains [33,36,37]. Recently, a method for ISSR-PCR identification of fungal strains was developed [38] that allows us to avoid the potential impact of resistance genes on the host and prevent generation of heterokaryons and mixed cultures. Finally, protoplast fusion has been used for mycovirus transmission [39], as purified VLPs are exploitable for transfecting protoplasts of virus-free strains [29,40].

Interestingly, the previous introduction of Sclerotinia sclerotiorum partitivirus 1 (SsPV1/WF-1) purified VLPs into virus-free *Botrytis cinerea* resulted in decreased mycelial growth and increased conidial production [41]. In this study, the conidial yields of Mm/MmPV1 were decreased >50% as compared to Mm, due to repressed expression levels of several conidiation-related genes, including *brlA* and *abaA*, two asexual developmental activators required in *Aspergillus* and insect pathogens of Hypocreales [42–44]. The sensitivity of Mm/MmPV1 to heat shock and UV-B correlated with reduced expression of genes involved in response to heat shock (*hsp20*, *hsp40a*, *hsp40b*, *hsp60* and *hsp70b*) and DNA damage repair genes (*asf1*, *PCNA*, *rad52*, *rad53*, *ku70*, and *top1*).

Furthermore, partitivirus infection may mediate host virulence. The alphapartitivirus Rhizoctonia solani partitivirus 2 (RsPV2) confered hypovirulence to the phytopathogenic fungus *Rhizoctonia solani* [45], while Botrytis cinerea partitivirus 2 (BcPV2) was also associated with host hypovirulence [46]. By contrast, the gammapartitivirus Talaromyces marneffei partitivirus-1 (TmPV1) enhances virulence due to upregulation of genes encoding γ-aminobutyric acid transaminase, nitrite reductase and nitrate transporters and downregulation of genes associated with RNA interference (RNAi) [40]. Our results reveal a link between MmPV1, host hypovirulence and transcriptional suppression (Fig 6). Several virulence-related genes, which contribute to conidial hydrophobicity and adhesion, are vital for initial cuticle infection, while secretion of cuticle-degrading enzymes is essential for successful cuticle infection [47,48] (Fig 7). This link provides a novel insight into the significance of MmPV1 infection for the virulence of *M*. *majus*.

Mycoviruses alter primary and secondary host metabolism. Antiviral fungal metabolites are markedly induced following infection with Cryphonectria hypovirus 1 [49]. Ochratoxin A, the second most important contaminant of food and feed, was overproduced when the host was infected with a partitivirus Aspergillus ochraceus virus [50]. Our study revealed the effect of mycovirus on the metabolome. The production levels of 22 major metabolites were significantly altered following MmPV1 infection. Compounds 20 and 21 were similar to the putative antiviral triterpenoids of *G*. *lucidum*, predicted to be viral protease inhibitors [51]. The attenuated virulence of Mm/MmPV1 could be associated with the absence of such compounds. The synthesis of metarhizins A and B, reported to have effective antiproliferative activity in the insect hemocoel [27], decreased significantly following MmPV1 infection. By contrast, MmPV1 increased the synthesis of nitrogen and phosphorus compounds involved in amino acid and nucleotide metabolic pathways. These results implicate MmPV1 in multiple metabolic pathways.

Additionally, heterologous expression of some viral proteins in fungi resulted in host morphological changes [52,53]. *M*. *majus* expressing MmPV1 ORF1 or ORF2 in showed no alteration of the phenotypes examined. Similar results were observed in four strains expressing a single viral protein from Fusarium graminearum virus 1 [54]. The present and previous studies indicate that mycoviruses might alter host traits by orchestrating expression of host genes rather than through the effects of their singular virus gene products. For instance, p29, a papain-like protein protease of CHV1, acts as a RNA silencer to suppress RNAi machinery [55].

In conclusion, our study revealed that the novel mycovirus MmPV1 decreases *M*. *majus* fitness to its host and environment through the coordination of conidiation, stress tolerance, pathogenicity, and secondary metabolism. These findings have broadened our knowledge on the diversity and horizontal transmission of mycoviruses, and their effects on their host’s lifecycle.

## Materials and methods

### Fungal strains and culture conditions

The *M*. *majus* strains RCEF0578 (MmPV1-infected, designated as Mm/MmPV1) and RCEF0577 (virus-free, designated as Mm) were originally derived from a stick-insect cadaver collected in Anhui Province and were conserved in Research Center for Entomogenous Fungi of Anhui Agricultural University (RCEF). The fungal strains were cultured on potato dextrose agar (PDA) in the dark at 25°C [56]. Conidia were harvested with 0.05% (v/v) Tween-80, and filtered through sterile nonwoven fabric to obtain conidial suspensions. M. majus strains were then cultured on Sabouraud dextrose agar yeast extract (SDAY), containing 1% (w/v) peptone, 4% (w/v) dextrose, 1% (w/v) yeast and 1.5% (w/v) agar, and covered with sterile cellophane, in the dark at 25°C.

### dsRNA extraction

Fresh mycelia (500 mg) of *M*. *majus* strains cultured on SDAY were collected, and dsRNA was extracted using CF-11 cellulose (Sigma) as previously described [57]. Subsequently, dsRNA extracts were treated with S1 nuclease and DNase I (TaKaRa, Dalian, China) to remove host RNA and DNA. Purified dsRNAs were detected through electrophoresis on 1.5% (w/v) agarose gels.

### RNA-sequencing, assembly and analysis

The dsRNA samples were sent to Illumina HiSeq 2500 platform at BGI (Shenzhen, China) for sequencing. The raw reads were processed using SOAPnuke (v1.4.0). The processed reads were aligned to the fungal genome using Bowtie2 (v2.2.9); the unaligned reads were assembled by Trinity (v2.1.1) for investigation of their coding capacity and quantification. The resulting thousands of contigs were queried against the non-redundant protein database at NCBI (https://www.ncbi.nlm.nih.gov) using BLASTx.

The 5′- and 3′-terminal sequences were obtained by RNA ligase mediated rapid amplification of cDNA ends (RLM-RACE) [58]. PCR amplicons were cloned into pMD18-T, transformed into *Escherichia coli* and sequenced at least three times. The sequences of MmPV1 dsRNAs 1 and 2 were analysed using ORF Finder (https://www.ncbi.nlm.nih.gov/orffinder) for open reading frame (ORF) prediction. Multiple sequence alignments were performed using MAFFT [59]. Phylogenetic analysis was performed using the maximum-likelihood (ML) method, the LG+G+I+F model and 1000 bootstrap replicates as implemented by MEGA X [60].

### Horizontal transmission of MmPV1

The MmPV1-infected strain RCEF0578 was co-cultured with MmPV1-free strain RCEF0577 for MmPV1 horizontal transmission. Following contact between the two strains at the colony margin, mycelia were sub-cultured on PDA for another 5 days until sporulation [33] and single spores from RCEF0577 were isolated.

Due to no obvious phenotypic differences between *M*. *majus* strains RCEF0578 and RCEF0577 (S1A and S1B Fig), a genotyping assay based on inter-simple sequence repeats (ISSR) was employed to discriminate each strain prior to repeated co-cultivation. This procedure reveals variability and phylogenetic relationships among strains [61,62]. Horizontal transmission of MmPV1 was confirmed by successful isolation of dsRNA and RT-PCR amplification.

### Plasmid construction and fungal transformation

The 3 × FLAG was fused in frame with the N-terminus of the MmPV1 ORFs 1 and 2 and the constructs were introduced into the binary plasmid pDHt-SK-bar-PgpdA to generate respectively pDHt-SK-bar-PgpdA-ORF1 and pDHt-SK-bar-PgpdA-ORF2, which were transferred into *Agrobacterium tumefaciens* strain EHA105. Then ORF1- and ORF2-expressing RCEF0577 stains were obtained via *Agrobacterium tumefaciens*-mediated transformation (ATMT) according to previous descriptions [63], and verified by RT-PCR, RT-qPCR and immunoblotting.

### Phenotype assays

To measure growth rate of individual *M*. *majus* strains, 1 μL aliquots of 1×10^7^ conidia mL^-1^ suspensions were spotted on PDA, SDAY and quarter-strength SDAY plates and incubated for 14 days in the dark at 25°C. Subsequently, colonies were photographed and colony diameters were measured [64].

Conidia production was examined as previously described [65]. Briefly, 30 μL aliquots of 1×10^7^ conidia mL^-1^ suspensions from individual *M*. *majus* strains were spread on PDA plates and incubated for 14 days as above. Collected conidia were resuspended in 30 mL 0.05% (v/v) Tween-80 solution by vortex mixing for 20 min prior to counting conidia using a heamocytometer.

To evaluate chemical stress tolerance, 1 μL aliquots of 1×10^7^ conidia mL^-1^ suspensions were spotted on PDA plates amended with NaCl (1 M), H_2_O_2_ (6 mM) and Conge Red (2 mg mL^-1^) and incubated for 14 days as above. Subsequently, colony diameters were measured to calculate relative mycelial growth inhibition rate [65].

To assess conidial germination rate, 10 μL aliquots of 5×10^6^ conidia mL^-1^ suspensions were spread on PDA plates and germination was observed under a microscope (Olympus BX 51, Tokyo, Japan) every 2 h to estimate median germination time (GT_50_). For tolerance assays to heat shock and UV-B irradiation, aliquots of 5×10^6^ conidia mL^-1^ suspensions were respectively incubated at 46°C for 1 h [64] or exposed to UV-B irradiation (312 nm wavelength at 100 mJ cm^−2^) in a UV crosslinker (HL-2000 Hybrilinker, UVP, Upland, CA, USA) [66]; then the conidia were spread onto PDA plates and incubated as above for 24 h prior to examination and assessment.

### Bioassays of fungal virulence

To determine the virulence of each *M*. *majus* strain, *Galleria mellonella* larvae were used for bioassays following topical application or injection of conidia [67]. Larvae were either immersed in 2×10^6^ conidia mL^-1^ suspensions for 90 s or injected with 10 μL of 5×10^4^ conidia mL^-1^ suspensions, and then incubated at 25°C. Mortality was recorded every 12 h and the median lethal time (LT_50_) was estimated. Each group contained 25 larvae and three independent groups were used to assess the virulence of each strain.

### Assays for fungal virulence-related phenotypes

To assess conidial hydrophobicity, 1×10^7^ conidia mL^-1^ suspensions in phosphate buffer were made and mixed with paraffin oil, following phase separation. Oil in the upper phase absorbs hydrophobic substances not found in the aqueous lower phase. Conidia in both the upper and lower phases were counted to calculate the hydrophobicity rate.

Conidial adhesion assays were carried out according to previous reports [25]. Drops of 2×10^7^ conidia mL^-1^ suspensions were placed onto sterile plastic plates (35 mm) and incubated at 25°C for 8 h. The plates were then washed with 1 mL double distilled water three times prior to counting the remaining conidia to calculate the adhesion index.

For appressorium formation assays, 1 mL aliquots of 1×10^6^ conidia mL^-1^ suspension in MMGly (minimal medium amended with 1% (v/v) glycerol) were placed onto sterile plastic plates (35 mm) and cultured at 25°C for 24 h. Appressoria were evaluated and measured under a microscope as described previously [68].

For penetration assays, a cicada (*Cryptotympana atrata*) wing assay was carried out. The wings attached to PDA plates were each inoculated with 1 μL drops of 2×10^6^ conidia mL^-1^ suspension, incubated for 3 days and then removed. The PDA plates were incubated for another 4 days to measure the colony diameter [68].

### Reverse transcription and quantitative polymerase chain reaction (RT-qPCR)

RT-qPCR was performed to determine the expression of selected genes related to conidiation, heat shock, UV-B irradiation resistance and virulence. For conidiation related genes, total RNA was extracted from mycelium cultured on PDA at 25°C for 60 h; for genes related to heat shock and UV-B irradiation, the conidia underwent respectively heat shock and UV-B irradiation as described above and cultured at 25°C for 24 h, then subjected to total RNA extraction. For virulence related genes, total RNA was extracted from *G*. *mellonella* infected by fungus at 48 h post inoculation. Total RNA was extracted as described previously [65], then transcribed into cDNAs using HiScript III 1st strand cDNA synthesis kit (Vazyme, Nanjing China) for RT-qPCR using AceQ qPCR SYBR Green Master Mix (Vazyme, Nanjing, China) on Real-time PCR system (CFX Manager Software; Bio-Rad, Hercules, CA, United States) [69]. The glyceraldehyde 3-phosphate dehydrogenase (*GAPDH*) gene (MAJ_05279) was used as reference gene [70], and the relative expression was calculated using the 2^-ΔΔCT^ method [71].

### High performance liquid chromatography—High resolutions mass spectrometry (HPLC-HRMS)

All solvents used for extraction were analytical grade (Sinopharm Chemical Reagent Co., Ltd., Shanghai, China). HPLC grade methanol and formic acid were purchased from Tedia Company of China (Shanghai, China). HPLC-HRMS data were obtained using an Agilent 1260 HPLC tandem 6545 QTOF MS spectrometer. The strains were cultured on SDAY at 25°C in the dark for 5–7 days and 30 mg freeze-dried mycelium mixed with 2 mL methanol underwent ultrasonic irradiation for 1 h, then the mixture was centrifuged at 12000 r min^-1^ for 10 min to obtain the supernatant. The methanol extract was analyzed with an Agilent Poroshell 120 EC-C18 (2.7 μm, 3.0 × 100 mm) column, and the LC parameters were set as follows: injection volume, 5 μL; column temperature, 25°C; and flow rate, 0.3 mL/min. The mobile phase was composed of (A) 0.1% (v/v) formic acid in water and (B) 0.1% (v/v) formic acid in acetonitrile, and a gradient elution was conducted: 0–3 min, 5% B, 3–10 min, 5–50% B, 10–38 min, 50–100% B, 38–48 min, 100% B. The eluates were monitored with a PDA performing a full wavelength scan from 200 to 600 nm, and a HRMS with the following parameter settings: gas temperature, 350°C; drying gas, 10 L/min; nebulizer pressure, 45 psi; capillary voltage, 4000 V in positive mode and 3500 V in negative mode; fragmentor voltage, 215 V in positive mode and 170 V in negative mode; skimmer voltage, 60 V. Data acquisition was performed in the m/z range of 50–1700 Da. The eluants of the preparative HPLC were detected by HPLC-HRMS without a chromatographic column.

### Metabolite identification

The molecular formulae of the metabolites were calculated by MassHunter (Version B. 07.00) based on accurate mass and isotopic pattern recognition. Compounds were putatively identified by searching the molecular formulae against the in-house entomopathogenic fungi database and the Dictionary of Natural Products (DNP) (https://dnp.chemnetbase.com/). The known compounds were confirmed by UV/visible spectra whenever possible and verified by their elution order (polarity) and structural characteristics. Molecular formulae without corresponding compounds in the database were labeled as unknown compounds.

### Statistical analysis

All experiments were repeated three times. GraphPad Prism v7.0 and SPSS v23.0 were used for statistical analysis. Normality and homoscedasticity were determined by Kolmogorov-Smirnov test and Levene’s test. Student’s t-test or one-way analysis of variance (ANOVA) followed by a least significant difference (LSD) test were adopted to analyzed different experimental groups.

## Supporting information

S1 Fig The horizontal transmission of MmPV1. The hyphae (A) and fungal mats (B) of RCEF0578 (donor) and RCEF0577 (recipient) strain cultured in separate plate. (C) The ISSR primers screening, including M1, M10, M15, M17, P8, P9, P11, and P12. The left lane is the RCEF0578, and the right lane is the RCEF0577. (D) Confirmation of MmPV1 horizontal transmission in RCEF0577 strains with ISSR primer 889. M, DNA molecular weight marker; lane 1–4 indicate Mm/MmPV1-1, Mm/MmPV1-2, RCEF0577, and RCEF0578, respectively. (E) Confirmation of MmPV1 horizontal transmission in RCEF0577 strains with ISSR primer 850. M, DNA molecular weight marker; lane 1–4 indicate Mm/MmPV1-1, Mm/MmPV1-2, RCEF0577, and RCEF0578, respectively.
(TIF)

S2 Fig The verification of single spore. (A) The species identification of single spores using P9. (B) The dsRNA extraction of recipient *M*. *majus* strains.
(TIF)

S3 Fig Effect of MmPV1 on growth of *M*. *majus*. (A) Growth diameter of different strains cultured on PDA, SDAY and 1/4SDAY medium for 14days. (B) Colony morphology of different strains cultured on PDA, SDAY and 1/4SDAY medium for 14days.
(TIF)

S4 Fig Effect of MmPV1 on germination of *M*. *majus*. (A) The germination rate of different strains. (B) The median germination time (GT50) of different strains.
(TIF)

S5 Fig Effect of MmPV1 on chemical stresses of *M*. *majus*. (A) Growth diameter of different strains cultured on NaCl, Congo Red and H2O2 medium for 14days. (B) Colony morphology of different strains cultured on NaCl, Congo Red and H2O2 medium for 14days.
(TIF)

S6 Fig High resolution mass of the changed metabolites from the wild or virus deleted strain of *M*. *majus*. The number 1 to 22 are the metabolites changed more than 2 times; “+” and “-” means cation and anion.
(TIF)

S7 Fig Construction of different expression vectors and confirmation of different transgenic strains. (A) Construction of the vector used in this study. (B) RT-PCR of Mm-ORF1 and Mm-ORF2. (C) The Cq value of qRT-PCR of Mm-ORF1 and Mm-ORF2, GAPDH as the as reference gene. (D) Immunoblotting of Mm-ORF1 and Mm-ORF2.
(TIF)

S8 Fig Effect of overexpression mutant strains on conidiation of *M*. *majus*. Conidiation of different strains cultured on PDA medium for 14 days. ****, P <0.0001.
(TIF)

S9 Fig Tolerance of overexpression mutant strains to heat shock and UV-B irradiation. (A) The relative germination rates of different strains after heat shock treatment for 24 hours. (B) The relative germination rates of different strains after UV-B irradiation treatment for 24 hours. ****, P <0.0001.
(TIF)

S10 Fig Bioassays of overexpression mutant strains. (A) Survival rate of G. mellonella after injection of conidial suspensions of different strains. Control insects were treated with sterile water. (B) The mean lethal times to death (LT50) of different strains after injection. (C) Survival rate of G. mellonella after topical application of conidial suspensions of different strains. Control insects were treated with sterile water. (D) The mean lethal times to death (LT50) of different strains after topical application. ***, P <0.001.
(TIF)

S1 Table Information of the virus isolates used for sequence alignment and phylogenetic analysis of their RdRps in Fig 2. (DOCX)

S2 Table The germination percentages and numbers for Mm, Mm/MmPV1-1, and Mm/MmPV1-2 strains after heat shock and UV-B irradiation. (DOCX)

S3 Table Inter-simple sequence repeats (ISSR) primers for identification of Metarhizium majus strains. (DOCX)

S4 Table Primers for expressing vectors construction and PCR detection. (DOCX)

S5 Table The median lethal time (LT50) of Mm and Mm/MmPV1 strains. (DOCX)

S6 Table Paired primers used for transcriptional profiling of potential MmPV1-targeted genes in M.majus via qPCR. (DOCX)
