# Supplementary figures and images for: A novel partitivirus orchestrates conidiation, stress response, pathogenicity, and secondary metabolism of the entomopathogenic fungus Metarhizium majus
Source: PLoS Pathog. 2023 May 22;19(5):e1011397. doi: 10.1371/journal.ppat.1011397 (PMC10237674; doi:10.1371/journal.ppat.1011397)

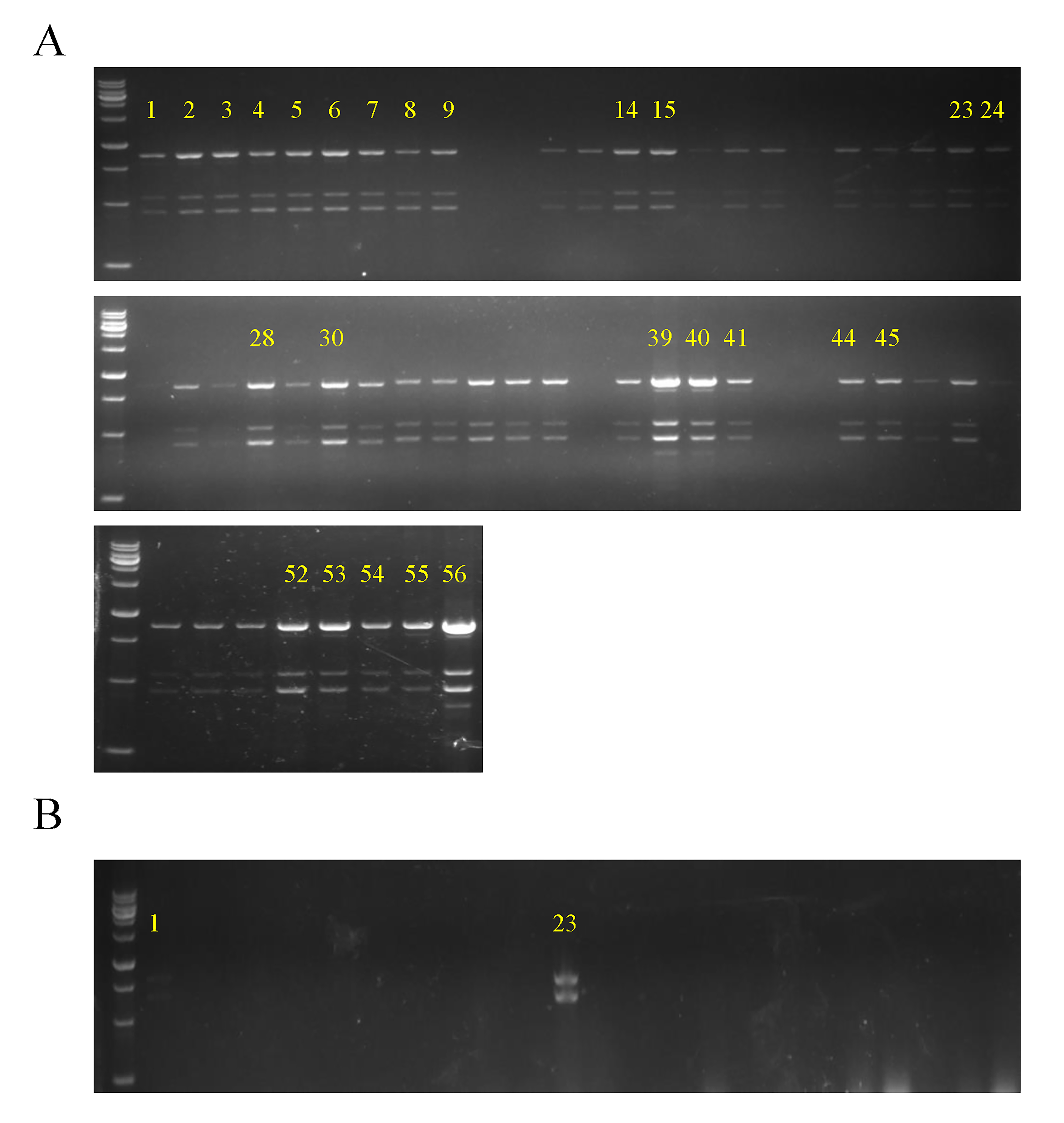

Supplement: S2 Fig — (A) The species identification of single spores using P9. (B) The dsRNA extraction of recipient M. majus strains. (TIF) [file ppat.1011397.s002.tif]

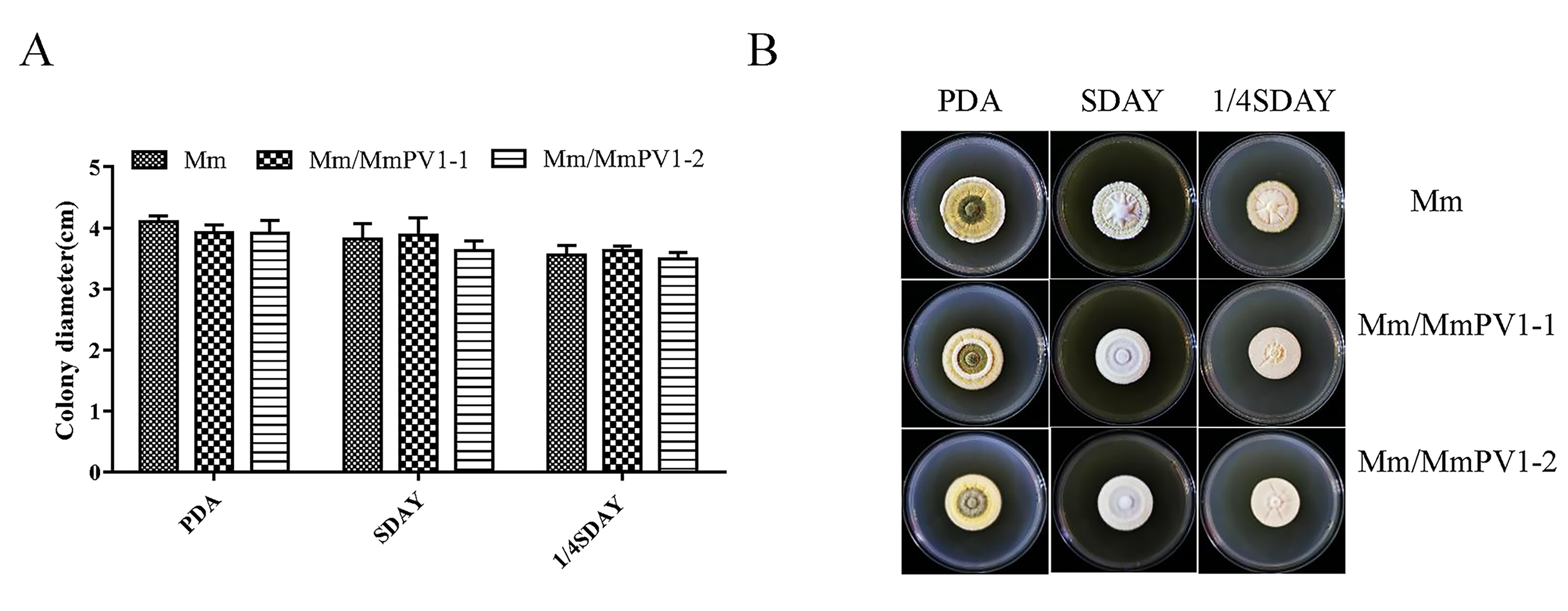

Supplement: S3 Fig — (A) Growth diameter of different strains cultured on PDA, SDAY and 1/4SDAY medium for 14days. (B) Colony morphology of different strains cultured on PDA, SDAY and 1/4SDAY medium for 14days. (TIF) [file ppat.1011397.s003.tif]

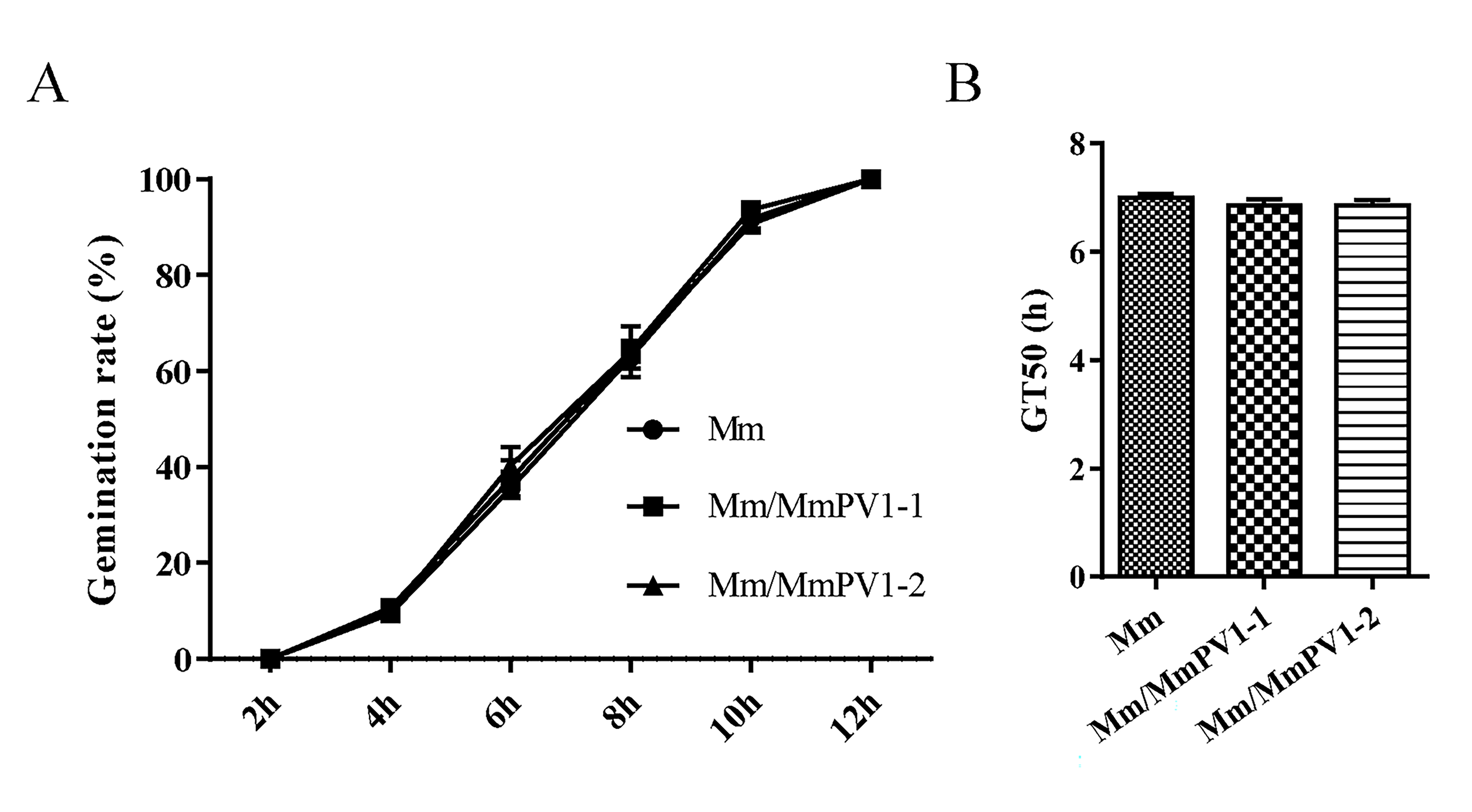

Supplement: S4 Fig — (A) The germination rate of different strains. (B) The median germination time (GT50) of different strains. (TIF) [file ppat.1011397.s004.tif]

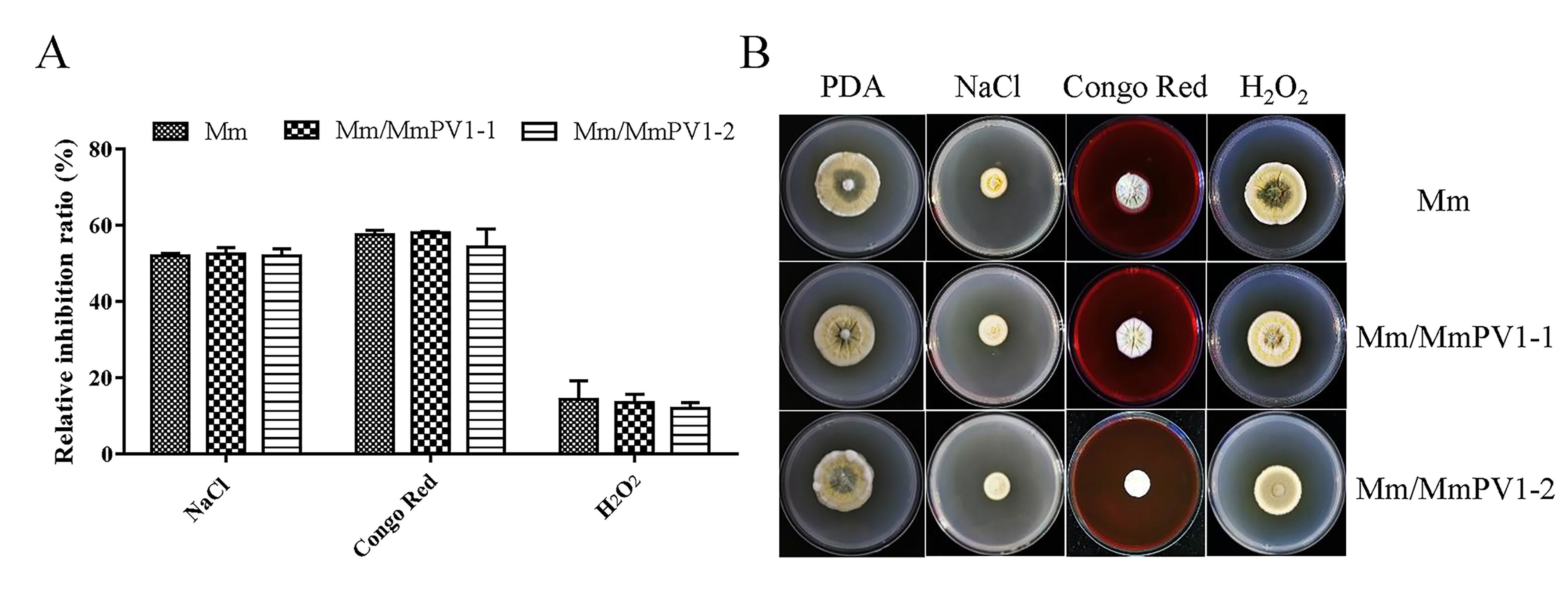

Supplement: S5 Fig — (A) Growth diameter of different strains cultured on NaCl, Congo Red and H2O2 medium for 14days. (B) Colony morphology of different strains cultured on NaCl, Congo Red and H2O2 medium for 14days. (TIF) [file ppat.1011397.s005.tif]

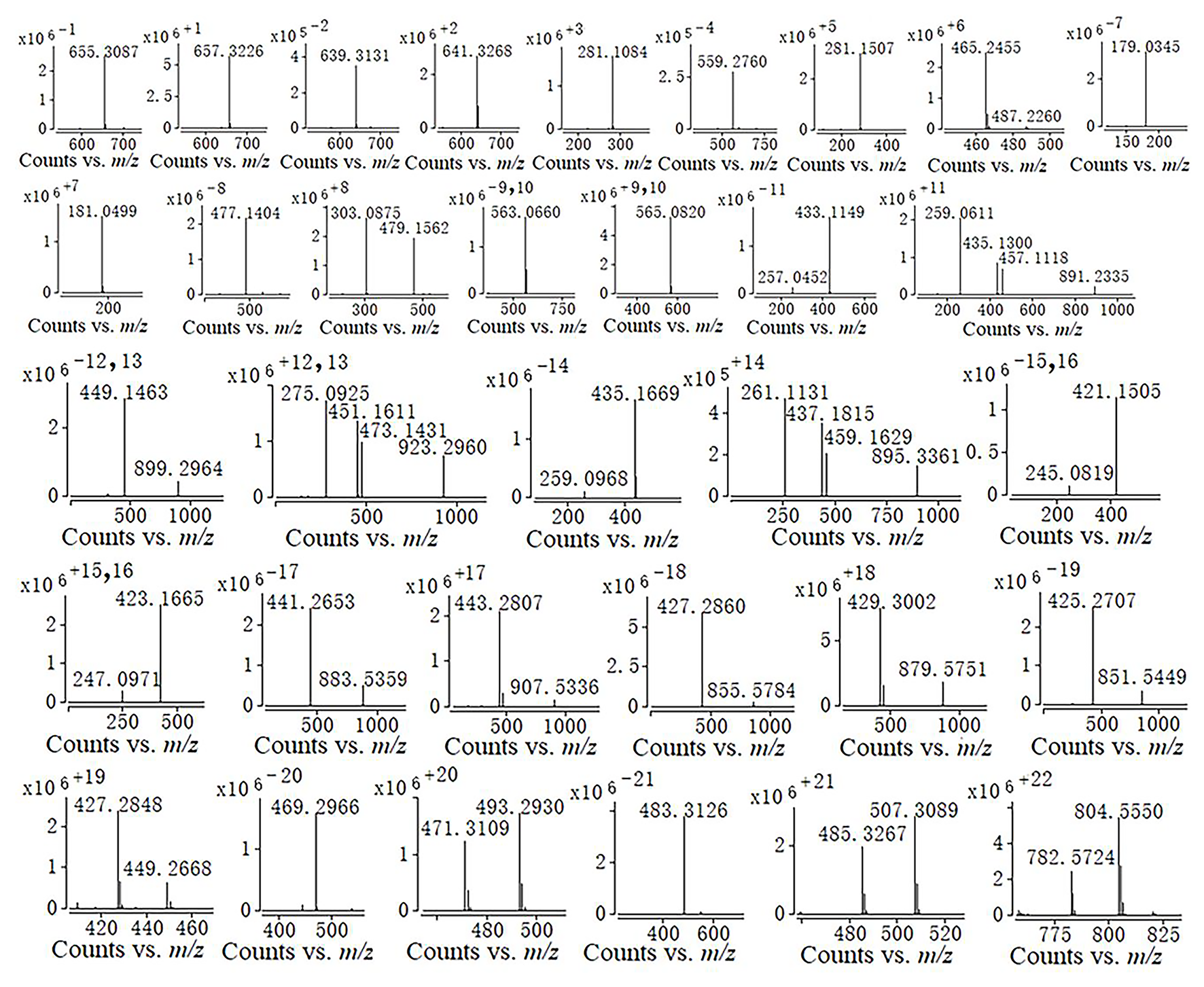

Supplement: S6 Fig — The number 1 to 22 are the metabolites changed more than 2 times; “+” and “-” means cation and anion. (TIF) [file ppat.1011397.s006.tif]

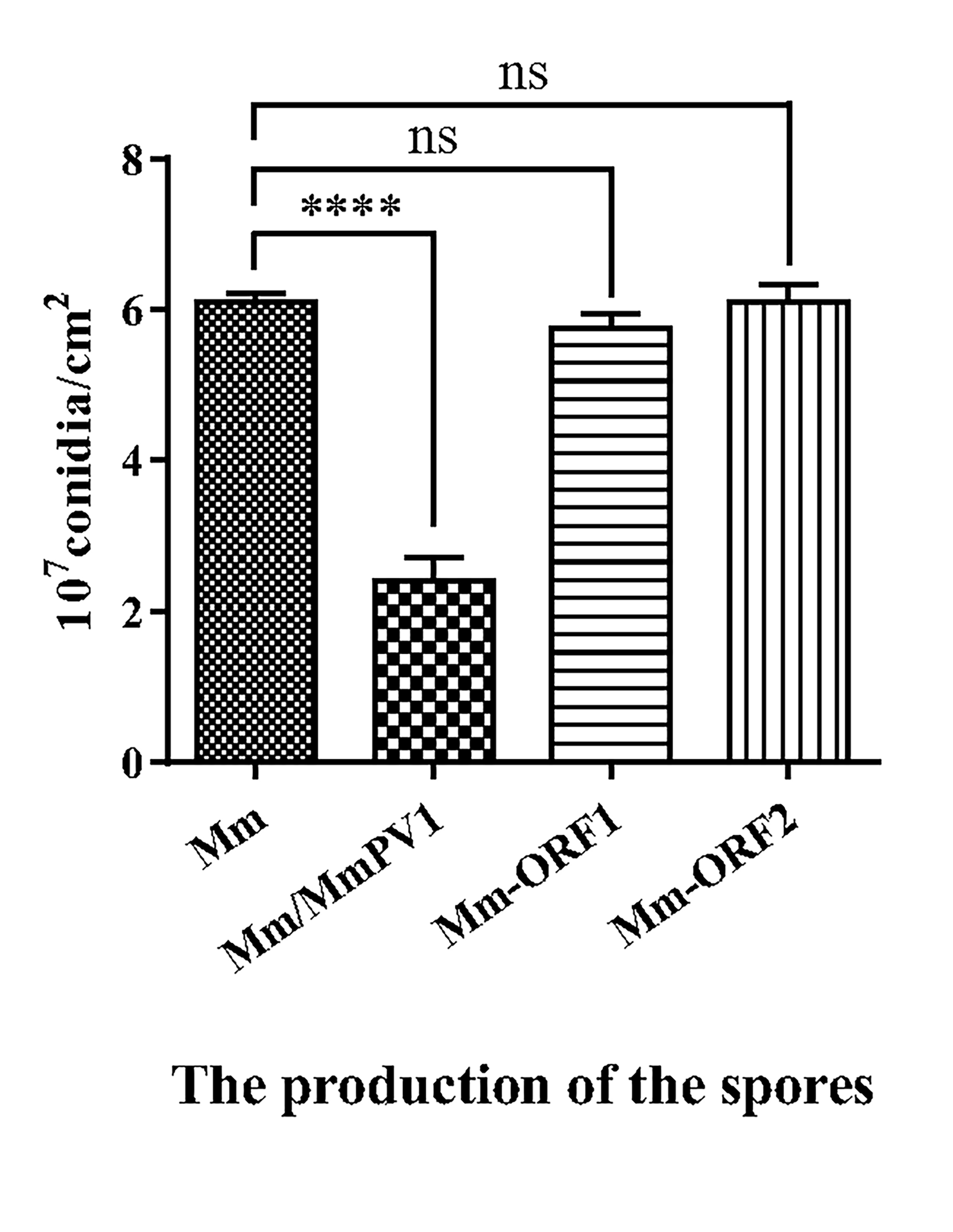

Supplement: S8 Fig — Conidiation of different strains cultured on PDA medium for 14 days. ****, P <0.0001. (TIF) [file ppat.1011397.s008.tif]
