# Supplementary material for: A novel partitivirus orchestrates conidiation, stress response, pathogenicity, and secondary metabolism of the entomopathogenic fungus Metarhizium majus
Source: PLoS Pathog. 2023 May 22;19(5):e1011397. doi: 10.1371/journal.ppat.1011397 (PMC10237674; doi:10.1371/journal.ppat.1011397)
Supplement: S1 Table — (DOCX) [file ppat.1011397.s011.docx]

**Table S1.** Information of the virus isolates used for sequence alignment and phylogenetic analysis of their RdRps in Fig. 2.

| **Virus name** | **Abbreviation** | **GenBank Acc.no.** | **F^a^** | **G^b^** |
| --- | --- | --- | --- | --- |
| Alternaria alternata partitivirus 1 | AtPV1 | APT70073.1 | Pa | Z |
| Aspergillus fumigatus partitivirus 1 | AfuPV-1 | CAY25801.2 | Pa | G |
| Aspergillus ochraceous virus | AoV | ABV30675.1 | Pa | G |
| Atkinsonella hypoxylon virus | AhV | NP_604475.1 | Pa | B |
| Beet cryptic virus 3 | BCV3 | AAB27624.1 | Pa | D |
| Botryosphaeria dothidea partitivirus 1 | BdPV2 | AGZ84316.1 | Pa | Z |
| Colletotrichum eremochloae partitivirus 1 | CePV1 | AZT88590.1 | Pa | E |
| Colletotrichum truncatum partitivirus 1 | CtParV1 | ALF46547.1 | Pa | G |
| Cryptosporidium parvum virus 1 | CSpV1 | AAC47805.1 | Pa | C |
| Discula destructiva virus 1 | DdV1 | AAG59816.1 | Pa | G |
| Discula destructiva virus 2 | DdV2 | AAK59379.1 | Pa | G |
| Fig cryptic virus | FCV | YP_004429258.1 | Pa | D |
| Fusarium solani virus 1 | FusoV | BAA09520.1 | Pa | G |
| Gremmeniella abietina RNA virus MS1 | GaRV-MS1 | AAM12240.1 | Pa | G |
| Heterobasidion partitivirus 1 | HetPV1 | YP_009508049.1 | Pa | A |
| Heterobasidion partitivirus 2 | HetPV2 | ADL66905.1 | Pa | B |
| Heterobasidion partitivirus 8 | HetPV8 | YP_009508063.1 | Pa | B |
| Magnaporthe grisea partitivirus 1 | MgPV1 | AZT88596.1 | Pa | G |
| Magnaporthe oryzae partitivirus 1 | MoPV1 | APP18151.1 | Pa | G |
| Magnaporthe oryzae partitivirus 2 | MoPV2 | ATD50490.1 | Pa | G |
| Metarhizium brunneum partitivirus 1 | MbPV1 | QHB49873.1 | Pa | E |
| Metarhizium brunneum partitivirus 2 | MbPV2 | QTC11257.1 | Pa | G |
| Ophiostoma partitivirus 1 | OPV1 | CAJ31886.1 | Pa | G |
| Penicillium aurantiogriseum partiti-like virus | PaPLV1 | YP_009182157.1 | Pa | E |
| Penicillium brasilianum partitivirus 1 | PbPV1 | AZT88608.1 | Pa | Z |
| Penicillium stoloniferum virus F | PsV-F | YP_271922.1 | Pa | G |
| Penicillium stoloniferum virus S | PsV-S | YP_052856.2 | Pa | G |
| Pepper cryptic virus 2 | PCV-2 | YP_009351838.1 | Pa | D |
| Pyrus pyrifolia cryptic virus | PpCV | BAA34783.1 | Pa | D |
| Raphanus sativus cryptic virus 1 | RsCV-1 | YP_656506.1 | Pa | A |
| Rosellinia necatrix partitivirus 1-W8 | RnPV1-W8 | YP_392480.1 | Pa | B |
| Ustilaginoidea virens partitivirus | UvPV | AGO04402.1 | Pa | G |
| Verticillium dahliae partitivirus 1 | VdPV1 | YP_009164038.1 | Pa | G |
| White clover cryptic virus 1 | WCCV-1 | YP_086754.1 | Pa | A |
| Beauveria bassiana polymycovirus 1 | BbPmV-1 | YP_009352879.1 | Po | P |

^a^F, Family; Pa, *Partitiviridae*; Po, *Polymycoviridae*. ^b^G, Genus; A, *Alphapartitivirus*; B, *Betapartitivirus*; C, *Cryspovirus*; D, *Deltapartitivirus*; E, *Epsilonpartitivirus*; G, *Gammapartitivirus*; Z, *Zetapartitivirus*; P, *Polymycovirus*
