# Supplementary material for: A novel partitivirus orchestrates conidiation, stress response, pathogenicity, and secondary metabolism of the entomopathogenic fungus Metarhizium majus
Source: PLoS Pathog. 2023 May 22;19(5):e1011397. doi: 10.1371/journal.ppat.1011397 (PMC10237674; doi:10.1371/journal.ppat.1011397)
Supplement: S2 Table — (DOCX) [file ppat.1011397.s012.docx]

Table S2 The germination percentages and numbers for Mm, Mm/MmPV1-1, and Mm/MmPV1-2 strains after heat shock and UV-B irradiation

|  | Mm | | | Mm/MmPV1-1 | | | Mm/MmPV1-2 | | |
| --- | --- | --- | --- | --- | --- | --- | --- | --- | --- |
|  | Treatment with heat shock | | | | | | | | |
| Germination percentages and numbers | 55.81%  (96/172) | 53.16%  (84/158) | 56.72%  (76/134) | 29.61%  (53/179) | 33.33%  (59/177) | 35.64%  (72/202) | 28.69%  (35/122) | 32.74%  (57/113) | 33.65%  (29/107) |
|  | Treatment with UV-B irradiation | | | | | | | | |
| Germination percentages and numbers | 80.33%  (98/122) | 79.41%  (108/136) | 76.19%  (96/126) | 49.33%  (74/150) | 42.68%  (69/163) | 42.42%  (56/132) | 49.40%  (82/166) | 40.28%  (58/144) | 44.93%  (62/138) |
