# Supplementary material for: A novel partitivirus orchestrates conidiation, stress response, pathogenicity, and secondary metabolism of the entomopathogenic fungus Metarhizium majus
Source: PLoS Pathog. 2023 May 22;19(5):e1011397. doi: 10.1371/journal.ppat.1011397 (PMC10237674; doi:10.1371/journal.ppat.1011397)
Supplement: S3 Table — (DOCX) [file ppat.1011397.s013.docx]

**Table S3.** Inter-simple sequence repeats (ISSR) primers for identification of *Metarhizium majus* strains

| **Primers** | **Sequences** |
| --- | --- |
| M1 | (GTC)_6_ |
| M10 | CCA(GTG)_4_ |
| M15 | (AC)_8_YG |
| M17 | (TG)_7_ACG |
| P8 | (GAG)_4_GC |
| P9 | (GACA)_4_ |
| P11 | (AG)_8_C |
| P12 | TG(CA)_6_C |
| 889 | DBD(AC)_7_ |
| 850 | (GT)_8_YC |
