# Supplementary material for: A novel partitivirus orchestrates conidiation, stress response, pathogenicity, and secondary metabolism of the entomopathogenic fungus Metarhizium majus
Source: PLoS Pathog. 2023 May 22;19(5):e1011397. doi: 10.1371/journal.ppat.1011397 (PMC10237674; doi:10.1371/journal.ppat.1011397)
Supplement: S4 Table — (DOCX) [file ppat.1011397.s014.docx]

**Table S4.** Primers for expressing vectors construction and PCR detection.

| **Primers** | **Paired sequences (5'-3')** | **Products** |
| --- | --- | --- |
| MmPV1-1-F | caagaacctttaatcgaattcATGATGGAGTCTTTCGACTCCCC/ | full-length of ORF1 (1659 bp) |
| MmPV1-1-R | cggtcggcatctactctgcagTCAATCAAACGGTCGATCTCCA |  |
| MmPV1-2-F | caagaacctttaatcgaattcATGTCGTCAGACAACACACGCT | full-length of ORF2 (1290 bp) |
| MmPV1-2-R | cggtcggcatctactctgcagTTAGACCGGTGCCTTTCCG |  |
| ORF1-F | CCGGATGTCTGTGACAAGATGA/ | parital sequence of ORF1 (304 bp) |
| ORF1-R | CGAAATGGACGGAAGGTACCTC |  |
| ORF2-F | CGTGATGCAGGGACTTTGTTCA | parital sequence of ORF1 (385 bp) |
| ORF2-R | CGAGAGAACCGCAAATGTCATC |  |
