# Supplementary material for: A novel partitivirus orchestrates conidiation, stress response, pathogenicity, and secondary metabolism of the entomopathogenic fungus Metarhizium majus
Source: PLoS Pathog. 2023 May 22;19(5):e1011397. doi: 10.1371/journal.ppat.1011397 (PMC10237674; doi:10.1371/journal.ppat.1011397)
Supplement: S5 Table — (DOCX) [file ppat.1011397.s015.docx]

**Table S5.** The median lethal time (LT_50_) of Mm and Mm/MmPV1 strains.

| topical infection | | | | | | | | |
| --- | --- | --- | --- | --- | --- | --- | --- | --- |
| Mm | | | Mm/MmPV1-1 | | | Mm/MmPV1-2 | | |
| 7.49 | 7.32 | 6.42 | 10.14 | 9.66 | 11.96 | 10.25 | 9.66 | 10.29 |
| injection bioassay | | | | | | | | |
| Mm | | | Mm/MmPV1-1 | | | Mm/MmPV1-2 | | |
| 3.98 | 4.02 | 3.86 | 4.08 | 4.22 | 4.21 | 4.00 | 4.59 | 4.31 |
