# Supplementary material for: A novel partitivirus orchestrates conidiation, stress response, pathogenicity, and secondary metabolism of the entomopathogenic fungus Metarhizium majus
Source: PLoS Pathog. 2023 May 22;19(5):e1011397. doi: 10.1371/journal.ppat.1011397 (PMC10237674; doi:10.1371/journal.ppat.1011397)
Supplement: S6 Table — (DOCX) [file ppat.1011397.s016.docx]

**Table S6.** Paired primers used for transcriptional profiling of potential MmPV1-targeted genes in *M.majus* via qPCR.

| **Gene** | **Tag loci** | **Annotation** | **Sequences (5’-3’) of paired primers** |
| --- | --- | --- | --- |
| **Involved in conidial yield** | | | |
| brlA | MAJ_01216 | Transcription factor Fst12 | ATCAACTCGGCTCTGTCG/ TGGCGTTGCTGATGCTAT |
| AbaA | MAJ_02577 | Transcription factor AbaA | AATCAGGTGGCCTTAGCA/ TTGACGAGGAGTCGGAAC |
| FluG | MAJ_08821 | Developmental protein FluG | TATTCGAGCCGTCAAGCA/ AAAGGCATTTCTGAGTCCA |
| SakA | MAJ_06162 | Stress-activated MAP kinase | AAATCTTCGGCACAACCT/ ATCTACAACGCCAGTCAG |
| AcoB | MAJ_04859 | COP9 signalosome subunit 7 | TCCACCACAGCCGTTCCT/ GCTCGGCGAAGATGTAGGT |
| FadA | MAJ_03946 | G protein alpha subunit | GAACGCTTGGAGTGGAAA/ CGTGCGGAACGACTGAAT |
| MpkA | MAJ_08054 | Protein kinase-like domain protein | CCTATGGCATTGTCTGGTAA/ CTCGCTCGTCTGGTTGTT |
| hymA | MAJ_01544 | Conidiophore development protein hymA | GGAGTATGCCTAATGAGAAC/ CGCAAGGAGGTACAGTAAA |
| FlbC | MAJ_00908 | C2H2 finger domain protein FlbC | TTTCCAATCTACGACGACA/ AGTCCGATTGATGGTCTTC |
| SteC | MAJ_03534 | MAP kinase kinase kinase | TTCCGCAAGAATCACATCA/ TTCCCATCTCCTTCAACACT |
| StuA | MAJ_05255 | APSES transcription factor | TCTGCTGCCCAAGGAGTG/ GAAGTGGTAGGACCGAAGG |
| PhiA | MAJ_05699 | Secretion pathway protein Sls2/Rcy1 | CGGTGTTGCGTGCTGTTT/ AATAATCGAGAACGTGGTTGA |
| **Involved in heat shock** | | | |
| hsp20 | MAJ_00722 | HSP20-like chaperone | GGGAAGCAGACTTTGATTGG/  CGTCGCAGTGTCGGTGGTGA |
| hsp30a | MAJ_07627 | Heat shock protein 30 | AGCTACACCCGTCAAGCAGG/  CTCGTATGCGTCGTCCGTCT |
| hsp30b | MAJ_02171 | Heat shock protein 30 | GCCTCAGACCTTGGTTGTTC/  TCGTGTATTCTTGGGCTCCT |
| hsp40a | MAJ_01851 | Heat shock protein DnaJ | CGTAAGCAAATCAGCGTCTG/  TTGGGTCCTTGTTCGTATCG |
| hsp40b | MAJ_07536 | DnaJ domain containing protein | GTCTTGAGCGACGCCGAACT/  GCCACCGAAGAATCGACTGA |
| hsp60 | MAJ_05291 | Heat shock protein 60 | GGGCTACGGCTCTGTCCTCT/  CGCGTCCTTCTCACCTTGTG |
| hsp70b | MAJ_05323 | Heat shock protein 70 | ATCACTGTCCCTGCCTACTT/  GCGATGAGACCAGCGTCCTT |
| **Involved in DNA damage repair** | | | |
| asf1 | MAJ_05851 | Histone deposition protein Asf1 | GAACCACCTCCCAGCGTCAT/  CGGGTCAGTTACCGTCCATC |
| chk2 | MAJ_04025 | Checkpoint kinase 2-like protein | TCACCCTTTCGCTCACCAAT/  CCGATCTCCGAGGCTTCTTA |
| PCNA | MAJ_02257 | Proliferating cell nuclear antigen PCNA | GCCGCCCAGAACGAAGACGT/  TCGGAATGCCCAGATGCTCC |
| Rad52 | MAJ_01395 | DNA repair and recombination protein rhm52 | GCACTCCCTCTGGTCTTCTG/  ACTCGTGGTTTAGCCTCCTC |
| Rad53 | MAJ_00832 | Serine/threonine-protein kinase domain protein | TCATTTGCGTGCTGTACCCG/  ATCATCCGCCTCGTTCTTGC |
| tel1 | MAJ_02739 | Phosphatidyl inositol 3-kinase | CAGCTCCGCTAGGTCAGAAT/  GAGGACTTGGTAGAGGTCGA |
| ku70 | MAJ_02874 | Ku70 protein | CTAGACATACAAGGCACAAA/  TAAGCATAGAGTCGCTAACT |
| top1 | MAJ_04070 | Topoisomerase I | AATGGTCCAGTTGATGATGC/  GAAGAGGAAGAAAGGCTATG |
| **Involved in cuticle degradation and virulence** | | | |
| hyd1 | MAJ_03467 | Hydrophobin | GGTGATGCCAACGCCCAGTG/  CCAGAAGGCCGTCGAGGACA |
| Mad1 | MAJ_03757 | Adhesin protein Mad1 | TCTGCTACCTTCGGCCTCTT/  TCACTCCAGTCCCAACCATT |
| Mad2 | MAJ_03792 | Adhesin protein Mad2 | CTGCCTGGGTCTTCATCTCA/  TTGTCGTCACAGCCGTCCAT |
| Mpl1 | MAJ_06865 | Perilipin MPL1-like protein | GGCTGTCCCTCAGGTCAATG/  GGCGAGGAGGTGCTGTAAAA |
| pr1A | MAJ_04022 | Subtilisin-like protease Pr1A | CCGTATCGCTACCGATGACA/  ATGGAAGGCGTGCTCGTAAA |
| pr1C | MAJ_07169 | Subtilisin-like serine protease PR1C | GTTGTTCAAGGGCGTCTCGG/  CTTTGGGCTTGGGCATGTTG |
| gpa | MAJ_00172 | Glycerol-3-phosphate O-acyltransferase | CCAAGGACGATTCTACTCAC/  GACCAAGTTGCCCATCATAT |
| chit30 | MAJ_05663 | Chitinase CHIT30 | TCCATCAAGAACTGCCAAAG/  CCCTGAGCGTCCGAGTGTAG |
